# Integrative Taxonomy of *Pachygrontha* (Heteroptera: Pachygronthidae) in East and Southeast Asia Reveals New Insights Into Species and Group Delimitation

**DOI:** 10.1002/ece3.73679

**Published:** 2026-07-01

**Authors:** Kaibin Wang, Cuiqing Gao, Ying Wang, Siying Fu, Wenjun Bu

**Affiliations:** ^1^ College of Life Sciences, Nankai University Tianjin China; ^2^ Co‐Innovation Center for Sustainable Forestry in Southern China, College of Forestry and Grassland, Nanjing Forestry University Nanjing China; ^3^ School of Synthetic Biology, Research Institute of Applied Biology, College of Life Science, Shanxi University Taiyuan China

**Keywords:** integrative taxonomy, Lygaeoidea, mitochondrial–nuclear discordance, species delimitation, species group

## Abstract

Species discovery and description are fundamental to biodiversity documentation and evolutionary studies. Integrative taxonomy synthesizes morphological, genomic, and ecological evidence to provide a robust framework for testing species hypotheses and delineating boundaries in closely related lineages, thereby not only advancing taxonomic practice but also enabling critical evaluation of species group concepts. Here, we present a comprehensive assessment of the genus *Pachygrontha* Germar, 1838 in East and Southeast Asia using an integrative approach that combines detailed morphology with complete mitochondrial genomes and genome‐wide SNPs. Our study confirms the existence of eight species, including six previously recognized species and two new species described herein as *P. chuanxiensis* Wang & Bu, sp. nov. and *P. ruiliensis* Gao, Wang & Bu, sp. nov. Phylogenetic analyses reveal instances of mito‐nuclear discordance and provide insights into the utility and limitations of historically defined species groups within *Pachygrontha*. Through examining differentiation at both the interspecific and closely related species‐levels, our integrative analysis provides a foundational assessment of divergence levels within the genus and establishes a basis for future broader surveys and more comprehensive taxonomic revisions.

## Graphical abstract

This manuscript presents a comprehensive taxonomic revision of the insect genus Pachygrontha in East and Southeast Asia, addressing the critical need for robust species delimitation in closely related lineages. Our research employs an integrative approach, combining detailed morphological examination with complete mitochondrial genomes and genome‐wide SNP data to test species hypotheses and evaluate the historical concepts of species groups within the genus.

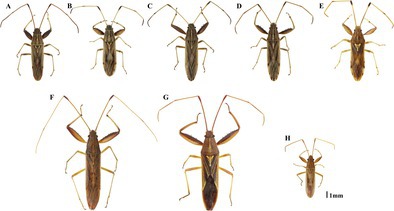

## 1 Introduction

Species discovery and description are fundamental for documenting biodiversity patterns and understanding the processes that drive organismal diversification (Schlick‐Steiner et al. 2010). Traditional taxonomy, which relies primarily on morphological characters, often faces limitations when dealing with taxonomically challenging groups, such as highly polymorphic species or complexes with minimal interspecific morphological differentiation (Struck et al. 2018). Over the past two decades, integrative taxonomy has emerged as a powerful framework, significantly advancing species discovery and delimitation by combining multiple sources of evidence (Dayrat 2005; Schlick‐Steiner et al. 2010; Singhal et al. 2025; Sites and Marshall 2003). This approach enhances and supplements traditional methods by integrating diverse data types (e.g., morphological, genetic and ecological) and methodologies (e.g., phylogenetic inference and ecological models). In doing so, integrative taxonomy effectively addresses long‐standing issues such as subjective interpretation and the limited resolution of single‐evidence systems, thereby increasing both the accuracy and efficiency of species delimitation (Schlick‐Steiner et al. 2010).

In addition to integrating evidence from multiple sources to support sound taxonomic conclusions and recommendations, integrative taxonomy can serve in practice as a framework for testing alternative hypotheses. Specifically, in the process of integrative taxonomic practice, analyzing the congruence and incongruence among different lines of evidence can help reveal the underlying evolutionary processes (Funk et al. 2021; Mason and Taylor 2015; Schlick‐Steiner et al. 2010). This approach elevates taxonomy beyond mere species naming to interpreting the evolutionary processes that generate diversity (DeSalle et al. 2005; Fujita et al. 2012; Smith and Carstens 2020). Such a perspective is particularly relevant for studying closely related species, which frequently assemble into distinct clusters or species groups. The differentiation among these groups reflects speciation dynamics shaped by particular biogeographic contexts or historical periods (Dal Vechio et al. 2020; Myers et al. 2020; Pallarés et al. 2024).

*Pachygrontha* Germar, 1838 (Hemiptera: Heteroptera: Pachygronthidae) represents the most species‐rich genus within the subfamily Pachygronthinae (Slater 1955), comprising approximately 70% of the known species in the subfamily (37 out of 53; species count according to the Lygaeoidea Species File, see Dellapé and Henry 2026). Multiple lines of evidence suggest an ancient origin for the genus, such as its unique transcontinental distribution spanning both Eastern and Western Hemispheres, and the presence of morphologically related yet geographically isolated forms across different continents without clear intermediate connections (Slater 1955). Previous studies have proposed various species combinations to describe this pattern, hypothesizing that several distinct species groups may reflect historical isolation events (Slater 1955; Wang et al. 2026). Typically, the delimitation of species groups relies on the degree of differentiation from other species within the genus. However, research on *Pachygrontha* remains largely dependent on Slater's 1955 study and has not incorporated newly described species since then (Slater 1955; Wang et al. 2026; Zheng et al. 1979). Other studies have mostly addressed taxonomic issues at the species level, overlooking the investigation of species groups (Aukema 2001; Hsiao et al. 1981; Schuh and Weirauch 2020). Furthermore, the taxonomic practice for distinguishing closely related forms frequently relies on relative morphometric measurements or subtle differences in coloration (Slater 1955), lacking corroboration from other data sources such as molecular evidence (as of March 2026, NCBI has only 25 sequences for *Pachygrontha*, most of which are from small markers like COI). Expanding research to include more species and integrating molecular data will help to better define species boundaries and provide a framework for the study of species groups.

East and Southeast Asia represent one of the regions with the highest species richness in this genus. By integrating morphological, mitochondrial genomic, and nuclear genomic data, we provide a comprehensive assessment of *Pachygrontha* species in this region, recognizing eight species, namely *Pachygrontha antennata* Slater, 1955, *P. similis* Uhler, 1896, *P. chuanxiensis* sp. nov., *P. flavolineata* Zheng, Zou & Hsiao, 1979, *P. ruiliensis* sp. nov., *P. nigrovittata* Stål, 1870, *P. semperi* Stål, 1870, *P. bipunctata* Stål, 1865. Our study reassessed species boundaries and evaluated the efficacy of both mitochondrial and nuclear markers in delimiting species and species‐group level relationships.

## 2 Materials and Methods

### 2.1 Sample Collection, DNA Extraction

**FIGURE 1:**
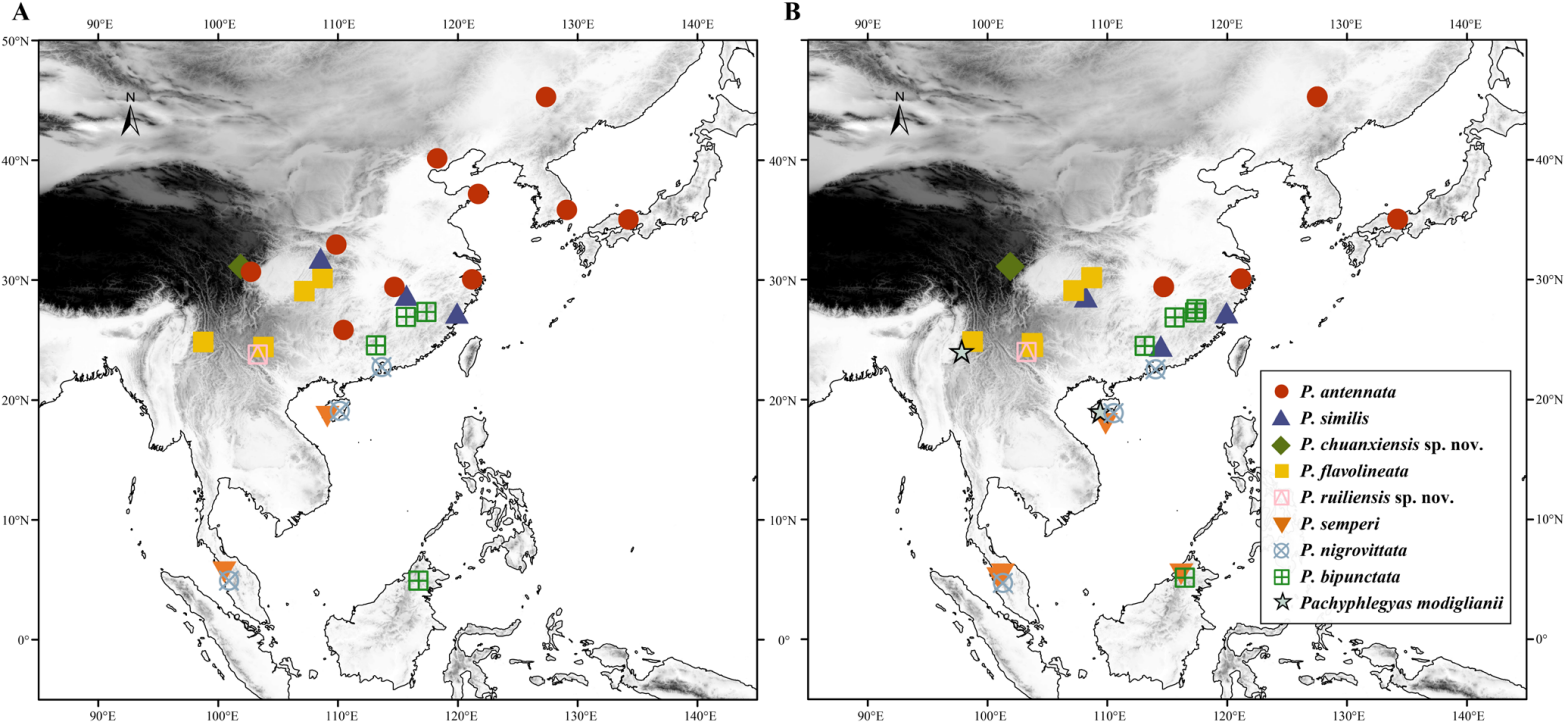
Sampling localities for mitochondrial (A) and nuclear (B) genomic analyses. Pentagrams denote outgroup taxa.

Mitogenomic analysis was performed using 46 specimens from *Pachygrontha* species, which contained 19 newly sequenced individuals. An outgroup was selected from within the same family, Pachygronthidae: *Stenophyella macreta* Horváth, 1914 (GenBank accession number: MW619646), a species that more closely resembles *Pachygrontha* in body size and morphology compared to *Pachyphlegyas* Slater, 1955 and *Opistholeptus* Bergroth, 1894. For genome‐wide sampling of single nucleotide polymorphisms (SNPs), the ddRAD libraries of 113 individuals were prepared according to Peterson's protocol (Peterson et al. 2012). The sample set included 41 newly sequenced individuals and four outgroup specimens of *Pachyphlegyas modiglianii* (Lethierry, 1889). Detailed information on specimens is provided in Figure 1, Tables S1 and S2. All previously sequenced individuals were derived from prior studies. The newly sequenced samples were initially preserved in anhydrous ethanol in the field and subsequently stored at −20°C. Genomic DNA was extracted from thoracic muscle tissue using a Universal Genomic DNA Kit (CWBIO, China). Voucher specimens have been deposited at the College of Life Sciences, Nankai University, Tianjin, China (NKUM).

### 2.2 Morphological Identification

Key morphological characters of all specimens were systematically observed and compared under a stereomicroscope. Based on these observations, species delimitation was primarily guided by the original descriptions, supplemented by additional literature (Hsiao et al. 1981; Slater 1955; Zheng et al. 1979).

### 2.3 Molecular Analysis of Mitochondrial Genomes

#### 2.3.1 Sequencing, Processing and Sequence Analyses

Library assembling and sequencing were conducted on the Illumina NovaSeq 6000 platform, generating 150 bp paired‐end reads. To ensure accuracy, the mitogenome was assembled using two complementary strategies. De novo assembly was performed with IDBA‐UD v1.1.3 (Peng et al. 2012), using k‐mer values ranging from 40 to 120 bp. Additionally, a reference‐based assembly was carried out with MITObim v1.9 (Hahn et al. 2013), in which clean reads were mapped to the reference mitogenome of *Stenophylla macreta*. The mitochondrial gene sequences were aligned using MAFFT v7.402 (Katoh and Standley 2013) under the G‐INS‐i strategy. Protein‐coding genes (PCGs) and ribosomal RNA (rRNA) genes were annotated by aligning with homologous sequences from the published mitogenome of *P. antennata* (GenBank accession number: OR134608).

#### 2.3.2 Dataset Establishment

After the removal of stop codons, mitochondrial gene alignments were concatenated using PhyloSuite v1.2.2 (Zhang et al. 2020) to construct two datasets: PCG dataset, which included all three codon positions of the 13 mitochondrial PCGs, and PCGR dataset, comprising both the 13 PCGs and two rRNA genes.

#### 2.3.3 Molecular Species Delimitation

We employed four delimitation methods to investigate species boundaries based on the PCG dataset: two distance‐based species delimitation methods, including Automatic Barcode Gap Discovery (ABGD) (Puillandre et al. 2012) and Assemble Species by Automatic Partitioning (ASAP) (Puillandre et al. 2021), and two tree‐based species delimitation methods, Bayesian Poisson Tree Processes (bPTP) (Zhang et al. 2013) and the Generalized Mixed Yule Coalescent (GMYC) model (Fujisawa and Barraclough 2013; Pons et al. 2006). ABGD analysis was performed on the ABGD web server (https://bioinfo.mnhn.fr/abi/public/abgd/) using the following parameters: prior intraspecific divergence values (*p*_min_ = 0.001 and *p*_max_ = 0.1), relative gap width (*X* = 1.5), and the Kimura 2‐P (K80) distance model. ASAP analysis was conducted on the website (https://bioinfo.mnhn.fr/abi/public/asap/) with the Kimura (K80) Ts/Tv 2.0 distance model. For bPTP analysis, we utilized a non‐ultrametric phylogenetic tree generated by IQ‐TREE as input on the bPTP web server (https://species.h‐its.org/ptp/), with outgroup sequences removed prior to analysis. GMYC analysis was conducted online (https://species.h‐its.org/gmyc/) using a single‐threshold method based on the ultrametric phylogenetic tree generated by BEAST 2.4.8 (Bouckaert et al. 2014).

#### 2.3.4 Phylogenetic Analyses

Phylogenetic relationships were reconstructed using three methods: Neighbor‐net (NN), Bayesian inference (BI), and maximum likelihood (ML). The NN analysis was conducted in SplitsTree v4.17.1 (Huson and Bryant 2006) based on genetic distances. Prior to tree inference, the best‐fit partitioning schemes and nucleotide substitution models were selected under the Bayesian Information Criterion (BIC) using PartitionFinder v2.1.1 (Lanfear et al. 2016). BI analysis was performed using MrBayes v3.2.7a (Ronquist et al. 2012), with two independent MCMC runs of 10 million generations each, sampling trees every 1000 generations and discarding the first 25% as burn‐in. ML analysis was conducted with IQ‐TREE v2.2.0 (Minh et al. 2020), employing 1000 bootstrap replicates under the optimal substitution model. Genetic differentiation among lineages was evaluated in MEGA v11.0 (Tamura et al. 2021) using Kimura‐2‐parameter (K2P) genetic distances based on the PCG dataset. In addition, we performed principal component analysis (PCA) using the R package “adegenet” (Jombart 2008).

### 2.4 Molecular Analysis of Nuclear Genomes

#### 2.4.1 Generating ddRAD‐Seq Libraries

For ddRAD‐seq, all DNA samples were first evaluated by 0.8% agarose gel electrophoresis and quantified. Each library was double‐digested using the restriction enzymes *Eco*RI and *Msp*I. The ligation products were then pooled and size‐selected (250–600 bp) using a Pippin Prep system (Sage Science). Sequencing was performed on the Illumina NovaSeq 6000 platform. Finally, demultiplexing and SNP calling were conducted with ipyrad v0.9.42 (Eaton and Overcast 2020), which assigned reads to individual samples according to their barcodes.

#### 2.4.2 Genetic Structure and Polymorphisms

PA70_Matrix dataset (containing 7983 SNPs and 260 USNPs, clustering threshold = 0.85 and each locus was required to be present in at least 70% of individuals) generated by ipyrad was used for downstream analyses. Genetic clustering and admixture were assessed using four complementary approaches. First, a phylogenetic network was reconstructed using the Neighbor‐Net algorithm based on pairwise uncorrected *p*‐distances, implemented in SplitsTree. Next, population structure was inferred with STRUCTURE v2.3.4 (Pritchard et al. 2000), with the putative number of genetic clusters (*K*) ranging from 1 to 11, each run with 10 independent replicates. The optimal *K* value was determined using the delta *K* method (Evanno et al. 2005) in STRUCTURE HARVESTER (Earl and vonHoldt 2012), and 10 runs were combined into one output in CLUMPP v.1.1.2 (Jakobsson and Rosenberg 2007). The results were displayed graphically in DISTRUCT (Rosenberg 2004). PCA and discriminant analysis of principal components (DAPC) were then carried out using the “adegenet” package in R.

Genetic diversity was assessed based on observed heterozygosity (*H*_O_), expected heterozygosity (*H*_E_), and nucleotide diversity (*π*_S_), calculated using Arlequin 3.5 (Excoffier and Lischer 2010). SNP‐based inbreeding coefficients (*F*) were estimated with Plink 1.90 (Purcell et al. 2007). Pairwise *F*st values between species were also computed using Arlequin 3.5.

#### 2.4.3 Phylogenetic Analyses

We estimated species trees using coalescent‐based methods, SVDquartets (Chifman and Kubatko 2014) as implemented in PAUP* v. 4.0a169 (Swofford 2002). SVDquartets uses SNP data to infer phylogenetic relationships between quartets of taxa under the multispecies coalescent (MSC) model and then assembles these quartets into a species tree. Branch support was assessed through 100 nonparametric bootstrap replicates.

## 3 Results

### 3.1 Morphological Identification

**FIGURE 2:**
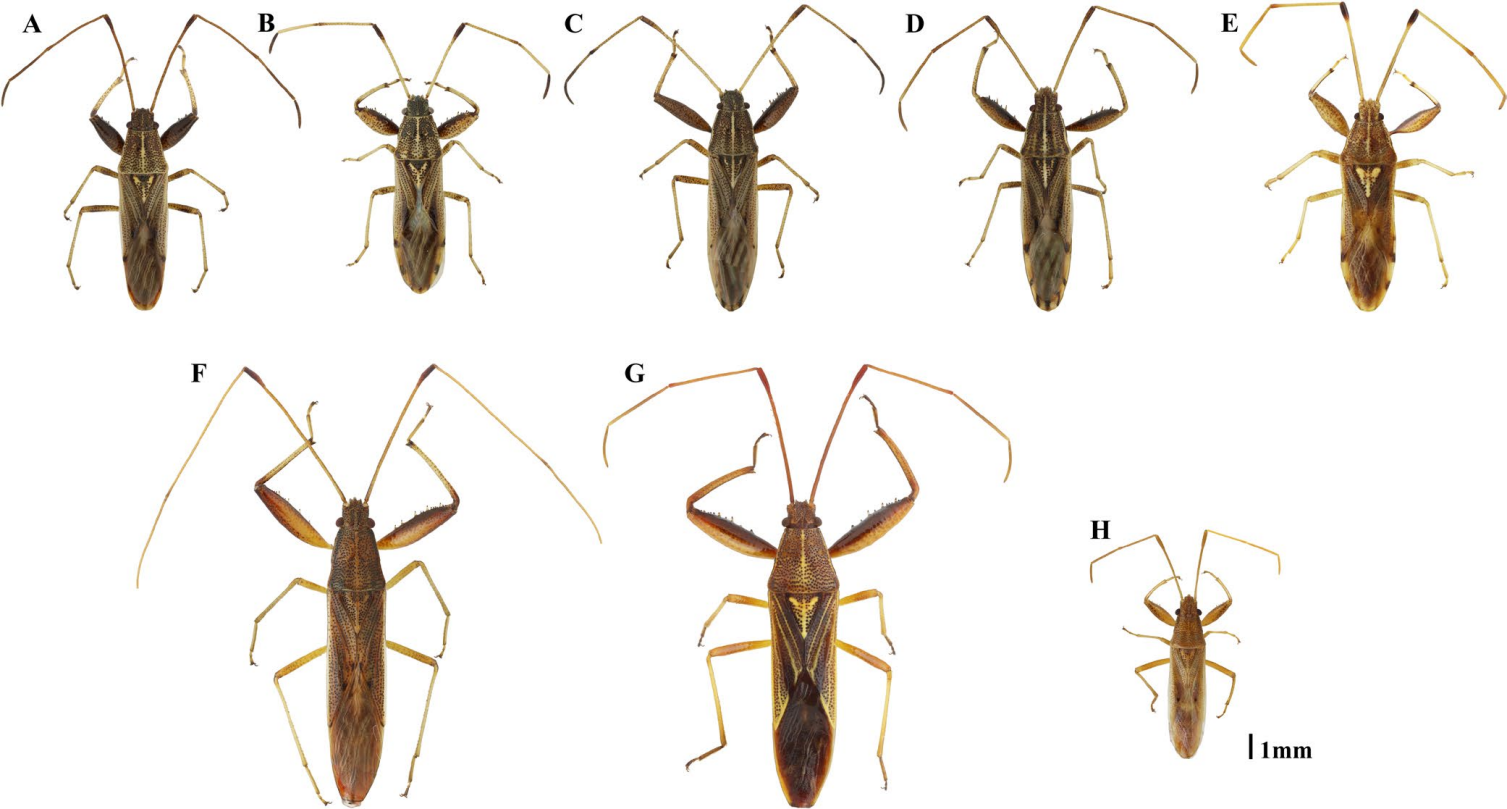
Dorsal habitus of male *Pachygrontha* species. (A) *P. antennata* . (B) *P. similis* . (C) *P. chuanxiensis* sp. nov. (D) *P. flavolineata* . (E) *P. ruiliensis* sp. nov. (F) *P. nigrovittata* . (G) *P. semperi* . (H) *P. bipunctata* .

All specimens were preliminarily identified and classified based on their original morphological descriptions, resulting in the recognition of six known species and two undescribed taxa (designated as SP1 and SP2) (Figure 2).

*P. nigrovittata* is relatively large in size, coloration ranging from brown to reddish‐brown. Pronotum flat, with median carina indistinct. Scutellum yellowish‐brown, flat, with tricarinate ridges weakly developed. In males, abdomen with black longitudinal bands laterally, and a black median stripe on the ventral surface of the last two segments. *P. semperi* is similar in body size to *P. nigrovittata* , coloration yellow to brown. Pronotum with a distinct yellow median vitta. Scutellum with a prominent yellow median carina, bifurcated near base to form two raised yellowish calli. Hemelytron with the median spot on the apical margin of corium extended anteriorly. Profemur deep reddish‐brown ventrally, yellow dorsally with brown spots. *P. bipunctata* is relatively small, pale yellowish‐brown. Pronotum with a broad, shallow transverse impression. Scutellum slightly elevated medially. Hemelytron lacking a spot at the apical angle of corium; median spot and small spot at apex of clavus brown. SP2 is yellowish‐brown, robust, and strongly shining. Lateral margins and median carina of pronotum yellow, with the latter fading before reaching the posterior margin. Transverse carina of scutellum distinctly elevated; the median carina together with the triangular area extending from the transverse impression to the apex of scutellum yellowish‐white, occupying most of the scutellar surface. Apical angle and median spot on apical margin of corium blackish‐brown. Abdominal venter yellowish‐brown, with lateral longitudinal bands blackish‐brown. *P. antennata* , *P. similis* , *P. flavolineata* and SP1 exhibit high similarity in body coloration, size, and maculation, suggesting that they may belong to the previously defined *the antennata group*. *P. flavolineata* pronotum strongly shining; median line and lateral margins smooth, carinate, pale yellowish‐brown, extending anteriorly to head margin and posteriorly to scutellum. Hemelytron with the median spot on apical margin of corium extended anteriorly into a blackish‐brown longitudinal band; apical angle of corium with a distinct black spot. Dorsal surface of connexivum pale yellowish‐brown, with each segment bearing a black spot at the posterolateral angle, or sometimes only the last two segments with such spots. Abdominal venter brown to blackish‐brown, with a broad median vitta and lateral bands black. SP1 is brown. Median carina of pronotum distinct on anterior lobe, gradually obsolete on posterior lobe. Corium with a very small spot at apical angle and a relatively small median spot on apical margin, the latter not or only slightly extended anteriorly. Abdominal venter reddish‐brown, with a fine, complete black median stripe. *P. similis* is yellowish‐brown and strongly shining. Pronotum constricted near posterior third, with a broad transverse impression; median carina distinct only on anterior lobe, obsolete on posterior lobe. Hemelytron with a broad blackish‐brown longitudinal band extending from the middle of the apical margin basally. Connexivum with a pair of distinct black spots on the dorsal surface of segments V, VI, and VII posteriorly. Abdominal venter brown, with lateral margins yellowish‐brown. *P. antennata* ranges from yellowish‐brown to dark brown. Pronotum yellowish‐brown, densely punctate, with lateral margins concave posteriorly. Hemelytron with apical angle, inner angle, and a large median spot on the apical margin blackish‐brown. Transverse carina of scutellum arched and elevated. Abdomen black dorsally, with connexivum yellowish‐brown; venter blackish‐brown, with a broad, complete black median stripe.

Although *P. antennata antennata* (Uhler, 1860) and *P. antennata nigriventris* Reuter, 1881 can be distinguished by relative proportions of morphological characters, transitional forms are present.

### 3.2 Molecular Species Delimitation Based on Mitochondrial Genomes

#### 3.2.1 Species Delimitation

**FIGURE 3:**
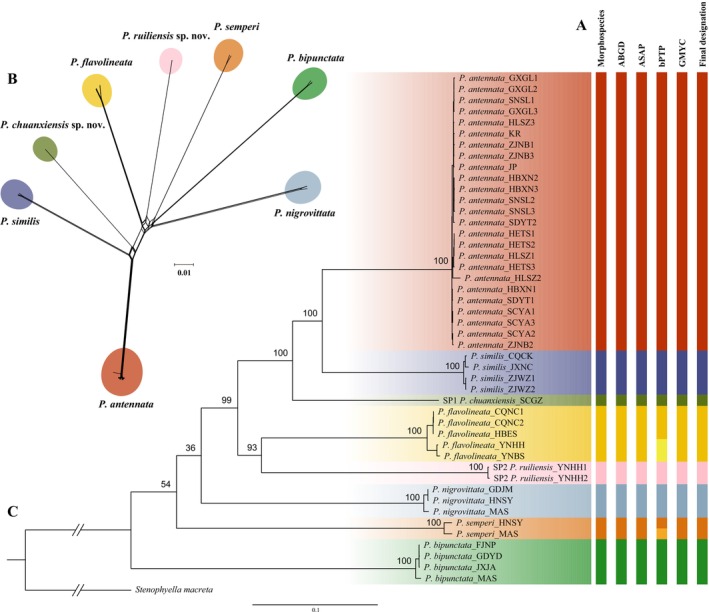
Phylogenetic relationships and species delimitation based on the PCG dataset. (A) Morphological identification and species delimitation results. (B) Phylogenetic network reconstructed using the neighbor‐net method. (C) Maximum likelihood phylogenetic tree.

The application of different approaches uncovered two distinct hypotheses for species delimitation. ABGD, ASAP, and GMYC analyses of the PCG dataset identified a total of eight molecular operational taxonomic units (MOTUs). The assignment of individuals to specific MOTUs was consistent with the phylogenetic clades, confirming the species status of the branch to be described (Figure 3A). Specifically, the distance‐based ABGD analysis consistently recognized eight MOTUs as the most robust result, with prior maximal distance (P) thresholds of 0.0010, 0.0016–0.0077, and 0.0129–0.1000 corresponding to 10, 9, and 8 MOTUs, respectively. ASAP also recovered eight MOTUs at a genetic distance threshold of 0.077017 (asap‐score 2.0, *p*‐value = 1.00e−05), whereas a threshold of 0.011914 (asap‐score 1.5, *p*‐value = 7.21e−02) resulted in nine MOTUs due to the division of *P. flavolineata* . In contrast, the tree‐based bPTP method supported a nine‐species hypothesis, with both *P. flavolineata* and *P. semperi* further split into two MOTUs each. Genetic distance analysis revealed that the net between‐group mean distance ranged from 0.129 to 0.202, and the lowest genetic distance was observed between (( *P. antennata* + *P. similis* ) + *P. chuanxiensis* sp. nov.) (Figure S1). The high differentiation among branches was also supported by the PCA results (Figure S2).

#### 3.2.2 Phylogenetic Analysis

All analyses based on mitochondrial data supported the monophyly of the eight morphologically identified species and the taxa awaiting formal description. The phylogenetic network based on the PCG dataset indicated clear boundaries between lineages (Figure 3B). However, phylogenetic trees reconstructed from different datasets (PCG and PCGR) and under different inference methods (ML and BI) exhibited conflicting topologies (Figure 3C, Figure S3). The clade comprising (( *P. antennata* + *P. similis* ) + *P. chuanxiensis* sp. nov.) was consistently recovered as a closely related and well‐supported complex, which together with ( *P. flavolineata* + *P. ruiliensis* sp. nov.) formed a larger species group. In contrast, the deeper phylogenetic relationships showed considerable inconsistency across analyses and were generally characterized by low nodal support. These topological ambiguities may result from limited resolution of mitochondrial markers at deep divergences and/or reflect insufficient sampling of extant species diversity (Ballard and Whitlock 2004; Nabhan and Sarkar 2012; Rubinoff and Holland 2005; Toews and Brelsford 2012).

### 3.3 Molecular Species Delimitation Based on SNP Data

#### 3.3.1 Population Genetics

**FIGURE 4:**
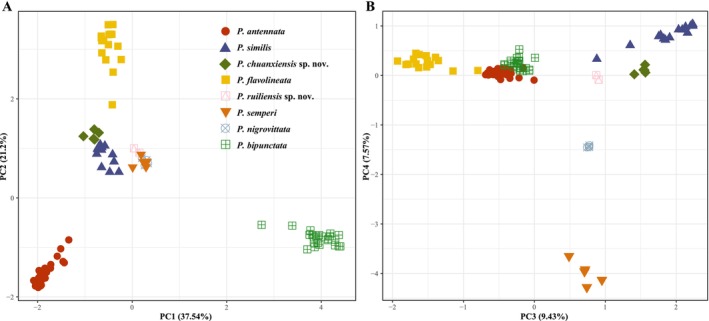
Principal component analysis (PCA) of the *Pachygrontha* species based on the PA70_Matrix dataset. Scatter plots show principal component combinations PC1 vs. PC2 (A) and PC3 vs. PC4 (B).

The phylogenetic network reconstructed from the PA70_Matrix dataset revealed deep divergence among lineages (Figure S4). The inferred genetic lineages were consistent with morphological and mitogenome‐based taxonomic assignments, with no evidence of transitional or hybrid individuals. STRUCTURE analysis identified *K* = 3 as the “optimal” clustering level, dividing the samples into approximately four branches. With the increasing of K values, further substructure emerged, ultimately recovering the eight lineages observed in the phylogenetic network at *K* = 9 (Table S3, Figure S5). This value also corresponded to the maximum mean LnP(K), indicating the best model fit. Consistent with the STRUCTURE results, PCA clearly separated *P. antennata* , *P. bipunctata* , and *P. flavolineata* along the first two components. Further differentiation was observed on PC4 (which explains 7.57% of the variance), where *P. ruiliensis* sp. nov., *P. semperi* , and *P. nigrovittata* were clearly distinguished (Figure 4). DAPC also reached a similar conclusion (Figure S6). Pairwise *F*_ST_ values between lineages ranged from 0.744 to 0.936, strongly supporting species‐level differentiation (Figure S7).

The genetic diversity results, including *F*, *H*_O_, *H*_E_, and *π*_S_ values are summarized in Table S4. Among all the lineages, *P. chuanxiensis* sp. nov. stands out as the most distinctive, characterized by notably low nucleotide diversity (0.0067 vs. 0.0154–0.0262 in other lineages) and a high inbreeding coefficient (0.9494 vs. 0.8622–0.9034). These genetic patterns may be attributed to its unique high‐altitude habitat (mainly found in the western Sichuan) and the relatively small effective population size (Wang et al. 2026).

#### 3.3.2 Phylogenetic Analysis

Phylogenetic trees based on nuclear genes show topological structures different from the mitochondrial genome. Although the combination of (( *P. antennata* + *P. similis* ) + *P. chuanxiensis* sp. nov.) still enjoys high support, *P. flavolineata* and *P. ruiliensis* sp. nov. have not been restored to sister species (Figure S8). In another large clade, the combination of (( *P. semperi* + *P. nigrovittata* ) + *P. bipunctata* ) did not appear in the results based on mitochondrial genomes either. The low support of some nodes also suggests that robust phylogenetic relationships between species and groups require more detailed sampling and more appropriate molecular marker selection in the future.

## 4 New Species Descriptions

**FIGURE 5:**
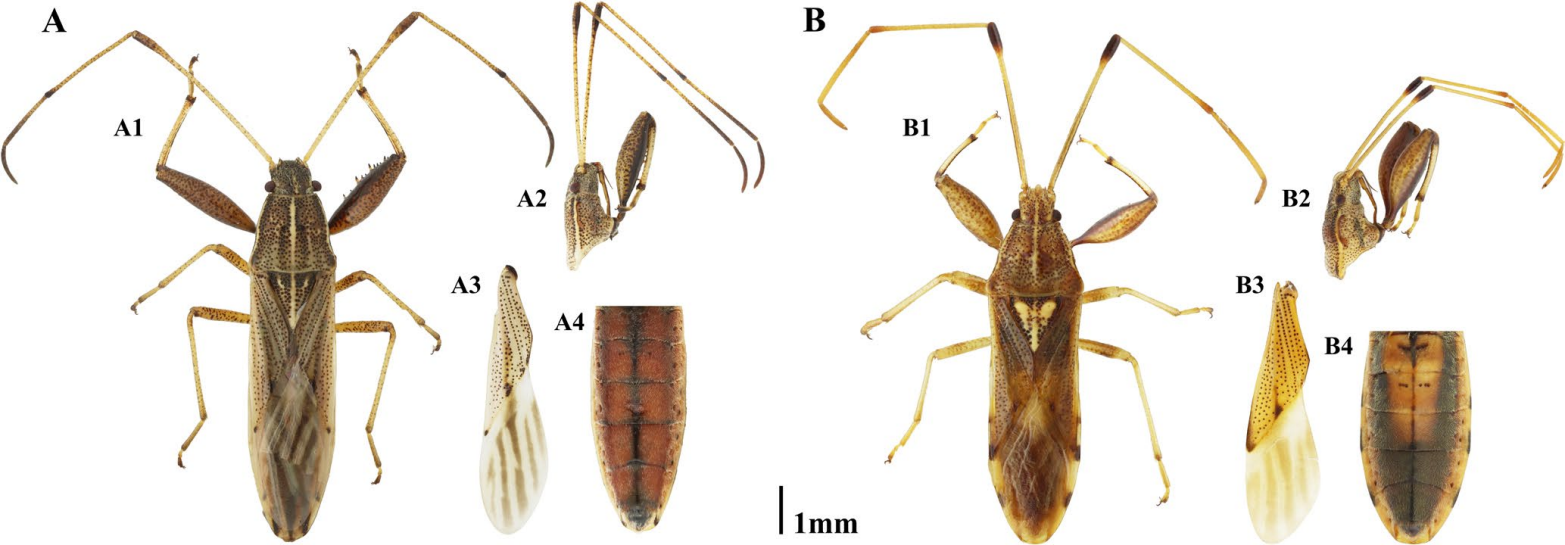
Dorsal habitus and morphological details of *P. chuanxiensis* sp. nov. (A) and *P. ruiliensis* sp. nov. (B). (A1, B1) Dorsal habitus; (A2, B2) head, lateral view; (A3, B3) forewing, dorsal view; (A4, B4) abdomen, ventral view.

Based on the integrative evidence presented above, we demonstrate the existence of two new species. Compared with other described species in the genus, they are morphologically and molecularly most closely related to *the antennata group*. These two species are described below (Figure 5).

**Pachygrontha chuanxiensis Wang & Bu, sp. nov**. (Figure 5A).

(urn:lsid:zoobank.org:act:96DB104A‐FEC9‐4990‐9C10‐6E7DD6B8074B).

Type material: *Holotype*. male, Barkam, Ngawa, Sichuan, China, 2800 m, 15 August 2022, Zechen Tang & Ying Wang (sweep net), (NKUM).

*Paratypes*. Same data as for holotype, 1 male (NKUM). Danba County, Ganzi Prefecture, Sichuan, China, 2600 m, 1 male, 4 nymphs, 14 August 2022, Zechen Tang & Ying Wang (sweep net), (NKUM).

Diagnosis: The new species is most similar to *P. flavolineata* in general body shape but is more elongate and slender. The transverse impression of the pronotum is shallow (Figure S9). The lateral margins of the pronotum bear a deep blackish‐brown longitudinal band on the inner side, with small, dense, black punctures on the anterior half, gradually becoming sparser posteriorly. The median carina of the pronotum is distinct only on the anterior lobe, becoming obsolete on the posterior lobe, yet it is longer than that of *P. similis* . The hemelytral corium bears very small markings at its apical angle and at the middle of the apical margin (Figure S10).

Structure Description: **Body** brown, covered with coarse, evenly distributed blackish‐brown punctations. **Head** dark brown, declivent, densely punctate; median carina yellowish‐white, extending to the posterior margin of jugal carina. **Antenna** yellowish‐brown with apex of antennomere I brown, and apices of antennomeres II and III, entire antennomere IV blackish‐brown. **Pronotum** with median carina extending to the posterior lobe, obsolete at its extreme terminus; lateral margin smooth, carinate, pale yellowish‐brown; paired opposing lunate markings flanking midline on anterior lobe, spots on posterolateral angles, and punctures black; posterior lobe bluish‐gray, each side with a smooth, slightly tumid longitudinal stripe, sparsely punctate on both sides of ridge. **Scutellum** with transverse ridge weakly tumid; median longitudinal carina extending from base to apex. **Hemelytra** brown, semi‐transparent; corium with apical angle and medial spot on apical margin distinctly small, black; membrane semi‐hyaline, brown. **Legs** pale grayish‐brown, scattered with tiny brown spots; fore femur incrassate, pale brown dorsally, black ventrally, armed below with 4–5 major spines and 7–8 minor spines; mid‐ and hind femora with small brown spots. **Thoracic venter** with broad longitudinal vittae on each sternite black, remaining areas reddish‐brown, punctate and clothed with dense, decumbent, silky setae; scent gland peritreme ear‐shaped, yellowish‐brown. **Abdomen** with black spots on apical angles of ultimate two sternites; sternites ventral midline black, running entire length.

Measurements: holotype (range of paratypes), mm. Body: length 8.08–8.22; width 1.85–1.89. Head: length 0.76–0.8; width 1.14–1.19. Length of antennomeres I–IV respectively 4.12–4.14, 2.52–2.53, 1.92–1.94, 1.16–1.17; Pronotum: median length 1.61–1.64; width at anterior margin 0.93–0.95; width at posterior margin 1.83–1.89. Scutellum: length 1.26–1.28; width 1.18–1.19. Length of fore femur 2.39–2.42.

*Etymology*. The specific epithet “***chuanxiensis***” is derived from Western Sichuan (Chuanxi in pinyin), China, which is the type locality of this species.

Distribution: China (Sichuan).

Note: The data for *P. chuanxiensis* sp. nov. included in this study were originally reported in a previous publication by our team (Wang et al. 2026). Here, we formally define this previously undetermined species based on a more comprehensive regional sampling context.

**Pachygrontha ruiliensis Gao, Wang & Bu, sp. nov**. (Figure 5B).

(urn:lsid:zoobank.org:act:A82DE692‐C189‐48C4‐AC87‐14F3AAE376FB).

Type material: *Holotype*. male, Ruili, Dehong, Yunnan, China, 29 July 2006, Xu Zhang (sweep net), (NKUM).

*Paratypes*. Same data as for holotype, 1 female, Weibing Zhu, 1 female, Ming Li (sweep net) (NKUM).

*Other material*. Mengzi, Honghe, Yunnan, China 2 female, 18 July 2023, Mu Qiao (sweep net), (NKUM).

Diagnosis: The new species shares a strong shiny body with *P. flavolineata* but is distinguished by its brighter, yellowish coloration, and relatively robust. Scutellum with yellowish area distinctly enlarged, nearly covering entire surface, bright yellow. Black spots are present on posterolateral angles of abdominal sternites IV‐VII (Figure S11).

Structure Description: **Body** robust, yellowish‐brown, shining. **Head** dorsum yellowish‐brown; median carina yellowish‐white, extending to the posterior margin of jugal carina. **Antennae** relatively short; antennomeres I–III yellow, with apex of antennomere I blackish‐brown; apex of antennomere II and base of antennomere III brown; antennomere IV entirely brown. **Pronotum** with a broad shallow transverse impression submedially; yellowish‐brown, sparsely covered with long setae; brown markings include paired, opposing lunate spots on anterior lobe adjacent to midline, spots on posterolateral angles, and punctures; lateral margin and median carina yellow, with latter fading posteriorly; calli strongly elevated; lateral margin sinuated at calli and transverse impression. **Scutellum** with a basal depression and a distinct pale Y‐shaped carina, the latter occupying most of scutellum. **Hemelytra** yellowish‐brown, semi‐transparent; inner margin of clavus and claval commissure reddish‐brown; apical angle of corium and a medial spot on apical margin blackish‐brown. **Membrane** brown, semi‐transparent, nearly reaching or slightly exceeding apex of abdomen. **Fore femur** incrassate, yellowish‐brown dorsally and covered with dark brown mottling, and reddish‐brown ventrally; armed below with 4–5 major spines and 7–8 minor spines, each with a conspicuous contrast between yellowish base and blackish‐brown apex. **Fore tibia** and **tarsus**, together with **mid** and **hind legs**, yellow; only claws of all legs darkened. **Mid‐** and **hind femora** occasionally with a few scattered dark punctures. **Thoracic pleura** yellowish‐brown, each with an obscure black longitudinal vittae lateral to supracoxal lobes; **thoracic sternites** black; **scent gland peritreme** ear‐shaped, yellow. **Abdominal connexivum** with posterior lateral angles of abdominal sternites IV‐VII black, with spot adjacent to corial apex being largest.

Measurements: holotype, mm. Body: length 8.26; width 2.48. Head: length 0.83; width 1.27. Length of antennomeres I–IV respectively 3.72, 2.42, 2.28, 1.21; Pronotum: median length 1.83; width at anterior margin 1.17; width at posterior margin 2.48. Scutellum: length 1.45; width 1.28. Length of fore femur 2.24.

*Etymology*. The specific epithet “*ruiliensis*” is derived from Ruili City in Yunnan Province, China, which is the type locality of this species.

Distribution: China (Yunnan).

## 5 Discussion

### 5.1 Species Delimitation

Although *Pachygrontha* is widely distributed across tropical and subtropical regions worldwide and has an early evolutionary origin (Slater 1955), molecular data for this genus remain scarce. This limitation has considerably constrained accurate assessments of species diversity and interpretations of speciation processes. In this study, we integrated multiple molecular markers and analytical approaches to delimit species boundaries of *Pachygrontha* in East and Southeast Asia. Our results consistently support the recognition of eight distinct species, including two newly described ones.

Analysis of mitochondrial genomes showed that genetic distances based on protein‐coding genes (PCGs) clearly separated species, with all interspecific divergences exceeding the empirical threshold of 3% (Hebert et al. 2003). Species delimitation results not only confirmed these eight species but also suggested further subdivision within *P. flavolineata* and *P. semperi* in bPTP analysis, elevating infraspecific lineages to species status. It should be noted that bPTP tends to exhibit oversplitting when applied to datasets containing multiple sequences (Luo et al. 2018; Pentinsaari et al. 2017). Considering established inter‐ and intraspecific genetic distance thresholds, the eight species hypothesis remains the best‐supported conclusion based on mitochondrial evidence. Analysis based on nuclear genomic data further confirmed the taxonomic status of the eight species. All genetic clusters were clearly delineated without evidence of transitional or hybrid individuals, reflecting deep evolutionary divergence and the maintenance of strong reproductive barriers. Although STRUCTURE analysis revealed shared ancestral components among some species (Figure S5), these components remained homogeneous and stable within each species, supporting their status as coherent evolutionary units.

At the subspecies level, intraspecific structure was detected in certain species only by the bPTP method based on mitochondrial genomic data, while no further subdivision was recovered by other methods or datasets. The subspecific structure revealed in previous studies focusing solely on *P. antennata* was not recovered here (Wang et al. 2025), possibly due to the inclusion of many distantly related species in our dataset, which may provide fewer informative sites for resolving intraspecific differentiation. In addition, due to sampling limitations, not all subspecies were included for some species (e.g., *P. bipunctata incipiens* distributed in South Africa), which affects our ability to assess the degree of subspecific differentiation within the genus *Pachygrontha*.

Collectively, the consistent results from both mitochondrial and nuclear datasets provide well‐supported species delimitation for *Pachygrontha* in East and Southeast Asia, confirming the utility of both marker types in species‐level identification. Future studies with more comprehensive sampling of *Pachygrontha* species and in‐depth investigation of their subspecies will contribute to a better assessment of global and regional diversity and help reveal the continuity of evolutionary processes from closely related species to infraspecific levels.

### 5.2 Phylogenetic Analysis and Species Group

While all species delimitation results consistently supported clear boundaries among species, phylogenetic relationships inferred from different molecular markers and analytical methods exhibited notable conflicts (Figures S3 and S8). Mitochondrial PCG‐based analyses identified *P. flavolineata* and *P. ruiliensis* sp. nov. as sister species, placed *P. bipunctata* in a basal position, and depicted deeper nodes as sequential divergences. In contrast, analyses incorporating both PCG and rRNA data (PCGR) suggested a sister relationship between *P. bipunctata* and *P. nigrovittata* , with *P. semperi* as the earliest diverging lineage (Figure S3). Nuclear genomic data further contradicted the mitochondrial topology by rejecting the sister relationship between *P. flavolineata* and *P. ruiliensis* sp. nov., instead supporting a clade composed of *P. nigrovittata* and *P. semperi* (Figure S8). The conflicts in phylogenetic relationships likely reflect both complex evolutionary relationships, such as incomplete lineage sorting or ancient hybridization (Avise and Robinson 2008; Sang and Zhong 2000; Whitfield and Kjer 2008), and the inherent limitations of different genomic markers, which may reflect the evolutionary history of their respective markers rather than the history of species differentiation (Maddison 1997; Nichols 2001; Toews and Brelsford 2012). Furthermore, the current limited taxon sampling may also contribute to the observed topological inconsistencies (Tamashiro et al. 2019).

East and Southeast Asia represent a major diversity center for *Pachygrontha*, hosting species belonging to Oriental‐Ethiopian, Palearctic, and Australian faunal elements, as well as five of the eight recognized species groups (the *bakeri group*, *the bipunctata group*, *the nigrovittata group*, *the antennata group*, *the lurida group*) (Table S5). Despite three of the four studied species groups being represented by only a single species, *the antennata group* (defined as comprising *P. antennata* and *P. similis* ) was consistently recovered as monophyletic across all analyses. Furthermore, integrative taxonomic approaches strongly support the monophyly of the clade comprising (( *P. antennata* + *P. similis* ) + *P. chuanxiensis* sp. nov.). Morphological and nuclear evidence also consistently position *P. flavolineata* as a closely related species. However, we have refrained from redefining *the antennata group* to include all four species ((( *P. antennata* + *P. similis* ) + *P. chuanxiensis* sp. nov.) + *P. flavolineata* ) for two primary reasons. First, mitochondrial data do not support this topology, instead recovering *P. flavolineata* as sister to *P. ruiliensis* sp. nov. This discrepancy suggests potential phenomena such as introgression or mitochondrial capture, making it inappropriate to treat these four species as a discrete group or complex isolated from other species. Second, species groups in this genus are traditionally established on morphological grounds, potentially reflecting common biogeographic or evolutionary histories to some extent (Slater 1955). *The antennata group* was originally conceived to represent a specific biogeographic scenario wherein *P. similis* was hypothesized to have dispersed from the mainland to Japan and speciated early, followed by a more recent divergence between the two subspecies of *P. antennata* . This geographically structured narrative, however, is inconsistent with the extensive sympatric distributions observed across the continent and the deep phylogenetic divergences centered in mainland regions rather than adjacent to island systems (Wang et al. 2025).

More extensive and broader geographical sampling of this genus may lead to a deeper understanding of its various species groups and help clarify the true utility of this taxonomic concept, both historically and in contemporary research. Studies built upon this foundation could establish a baseline for regional research, as this stable association may reflect a shared evolutionary history shaped by specific geological events or key historical periods. Focusing on such closely related species complexes provides a powerful framework for investigating microevolutionary processes—including hybridization, gene flow, and incomplete lineage sorting—without the confounding influence of deeper phylogenetic relationships.

## Author Contributions

**Kaibin Wang:** conceptualization (lead), resources (equal), software (equal), writing – original draft (lead). **Cuiqing Gao:** formal analysis (equal), funding acquisition (equal), resources (equal). **Ying Wang:** data curation (equal), resources (equal), software (equal). **Siying Fu:** funding acquisition (equal), software (equal), writing – review and editing (equal). **Wenjun Bu:** funding acquisition (equal), supervision (equal), writing – review and editing (equal).

## Funding

This work was supported by National Natural Science Foundation of China, 32130014, 32400359, 32572063.

## Conflicts of Interest

The authors declare no conflicts of interest.

## Supporting information


**Figure S1:** Heatmap constructed from estimates of pairwise mitochondrial genetic distance between species.
**Figure S2:** Results of Principal component analysis (PCA).
**Figure S3:** Phylogenetic tree inferred from the PCG dataset (Left) and PCGR dataset (Right). Values at nodes represent ML bootstrap/BI posterior probability.
**Figure S4:** The phylogenetic networks constructed by the neighbor‐net method based on the SNP dataset.
**Figure S5:** Results of structure analysis under *K* values from 1 to 11. Optimal clustering value for *K* = 3.
**Figure S6:** Result of Discriminant analysis of principal components (DAPC).
**Figure S7:** Heatmap constructed from estimates of pairwise *F*_st_ between species.
**Figure S8:** Inferred evolutionary relationships from SVDquartets analysis based on SNPs dataset.
**Figure S9:** Lateral view of head morphology in male *Pachygrontha* species. (A) *P. antennata* . (B) *P. similis* . (C) *P. chuanxiensis* sp. nov. (D) *P. flavolineata* . (E) *P. ruiliensis* sp. nov. (F) *P. nigrovittata* . (G) *P. semperi* . (H) *P. bipunctata* . The arrows indicate key diagnostic features, including: the depth of the transverse constriction of the pronotum, the morphology of the lateral carina, and the density and distribution of punctation.
**Figure S10:** Forewing view in male *Pachygrontha* species. (A) *P. antennata* . (B) *P. similis* . (C) *P. chuanxiensis* sp. nov. (D) *P. flavolineata* . (E) *P. ruiliensis* sp. nov. (F) *P. nigrovittata* . (G) *P. semperi* . (H) *P. bipunctata* . The arrows indicate key diagnostic features, including: the spots at the inner angle, apex, and middle of the apical margin of the corium, as well as the coloration between the veins of the membrane.
**Figure S11:** Ventral abdominal view of *Pachygrontha* species. (A) *P. antennata* , male. (B) *P. similis* , male. (C) *P. chuanxiensis* sp. nov., male. (D) *P. flavolineata* , male. (E) *P. ruiliensis* sp. nov., male. (F) *P. ruiliensis* sp. nov., female. (G) *P. nigrovittata* , male. (H) *P. semperi* , male. (I) *P. bipunctata* , male. The arrows indicate key diagnostic features, including: black markings on the connexivum adjacent to the apical angle of the corium, coloration of the abdominal venter, and the morphology of the mid‐ventral line.

**Table S1:** Information on sampling locations for mitochondrial analysis.
**Table S2:** Information on sampling locations for ddRAD analysis.
**Table S3:** Result of STRUCTURE Harvester under K values from 1 to 11.
**Table S4:** Nucleotide polymorphisms in each species based on SNPs dataset.
**Table S5:** Overview of major species groups within the genus *Pachygrontha* Germar, 1838, showing their distribution ranges and constituent species. Classification follows the system established by Slater (1955).

## Data Availability

The manuscript has been registered in ZooBank (urn:lsid:zoobank.org:pub:FA9C574F‐54A9‐4245‐8362‐827ED22A3E00). Newly sequenced mitogenomes were submitted to GenBank (accession numbers: PX583528 ‐ PX583546). Newly sequenced ddRAD data are available in the NCBI SRA (PRJNA1359458).
