# Supplementary material for: Integrative Taxonomy of Pachygrontha (Heteroptera: Pachygronthidae) in East and Southeast Asia Reveals New Insights Into Species and Group Delimitation
Source: Ecol Evol. 2026 Jul 1;16(7):e73679. doi: 10.1002/ece3.73679 (PMC13322632; doi:10.1002/ece3.73679)

**Integrative taxonomy of *Pachygrontha* (Heteroptera: Pachygronthidae) in East and Southeast Asia reveals new insights into species and group delimitation**

Kaibin Wang^1^†, Cuiqing Gao^2^†, Ying Wang^1^, Siying Fu^13^*, Wenjun Bu^1^*

^1^College of Life Sciences, Nankai University, Weijin Road, Nankai, Tianjin 300071, China

^2^Center for Sustainable Forestry in Southern China, College of Forestry and Grassland, Nanjing Forestry University, LongPan Road, Nanjing, Jiangsu 210037, China

^3^School of Synthetic Biology, Research Institute of Applied Biology, College of Life Science, Shanxi University, Taiyuan, Shanxi, 030006 China

†These authors contributed equally to this work.

*Correspondence to be sent to: College of Life Sciences, Nankai University, Tianjin 300071, China; e-mail: [wenjunbu@nankai.edu.cn](mailto:wenjunbu@nankai.edu.cn); [nkufsy@163.com](mailto:nkufsy@163.com)

Supplementary Figure 1. Heatmap constructed from estimates of pairwise mitochondrial genetic distance between species.
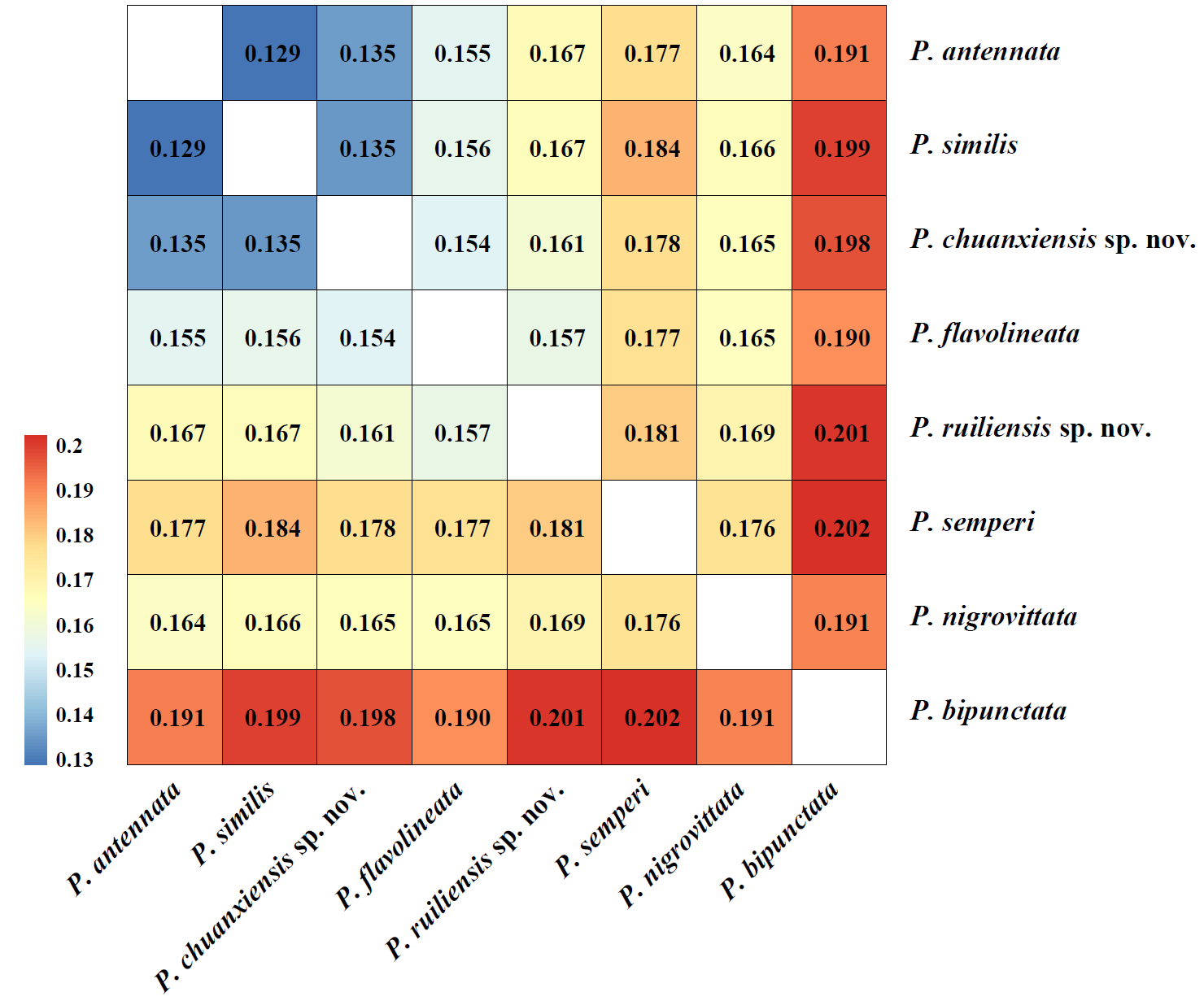


Supplementary Figure 2. Results of Principal component analysis (PCA).


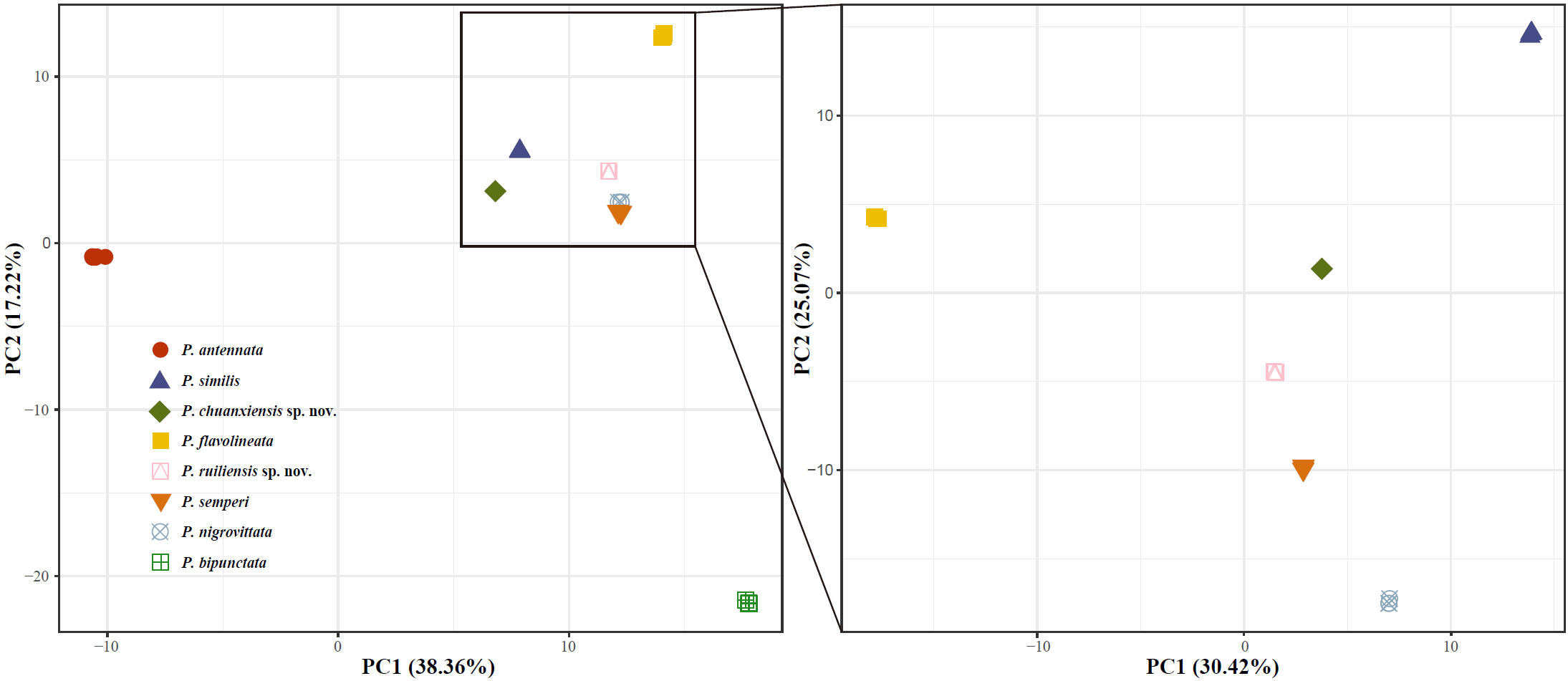


Supplementary Figure 3. Phylogenetic tree inferred from the PCG dataset (Left) and PCGR dataset (Right). Values at nodes represent ML bootstrap / BI posterior probability.


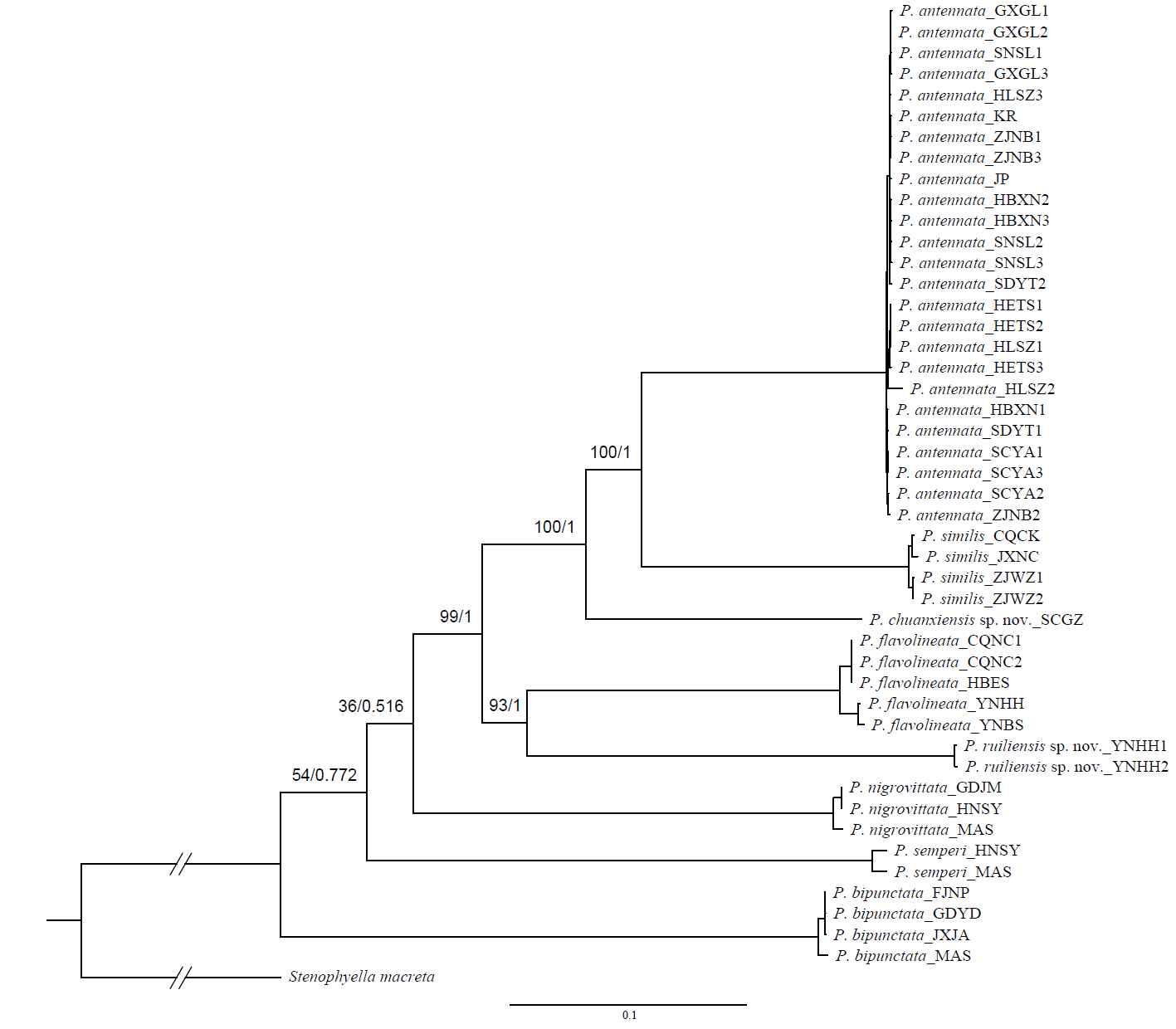

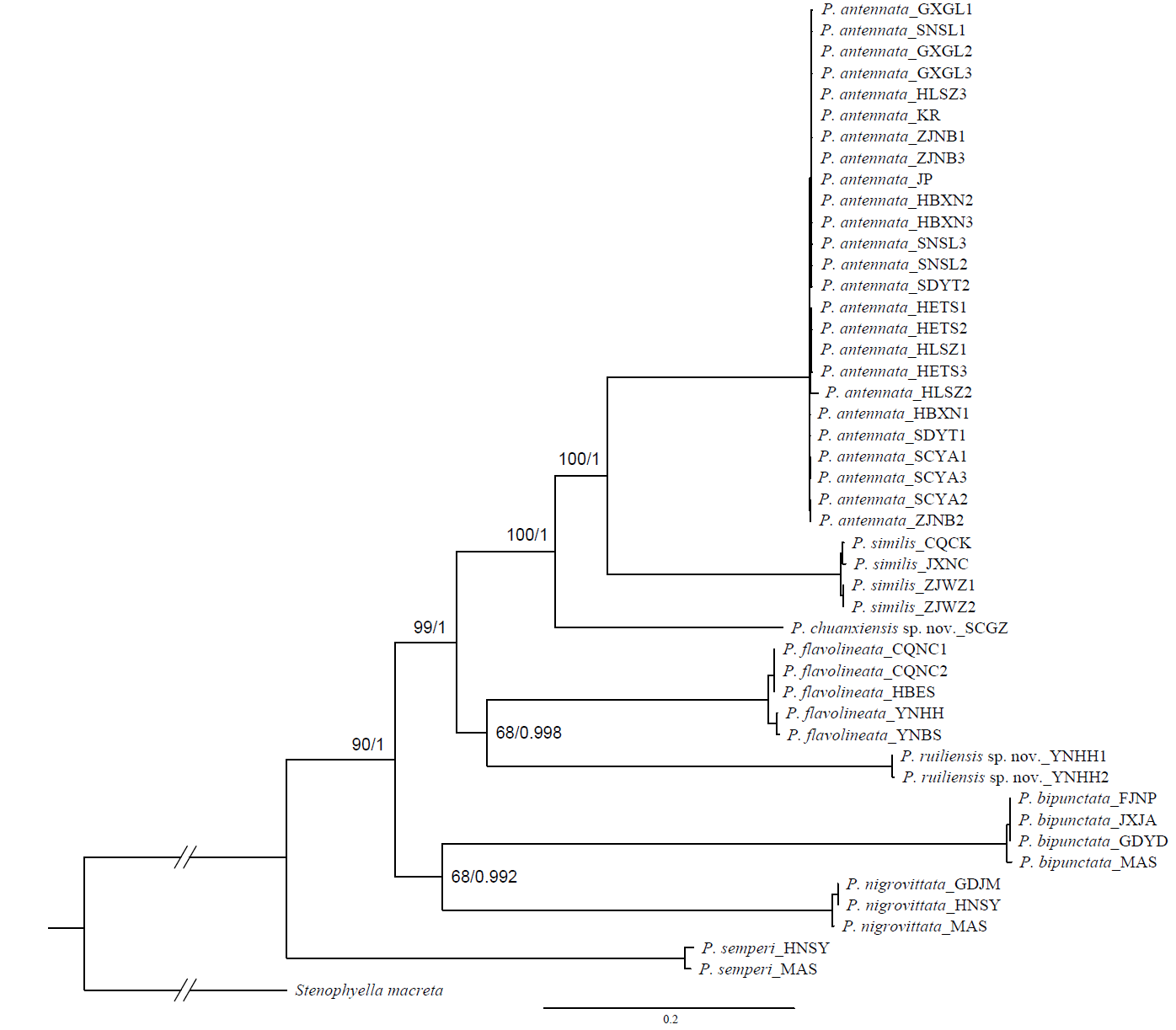


Supplementary Figure 4. The phylogenetic networks constructed by the neighbor-net method based on the SNP dataset.


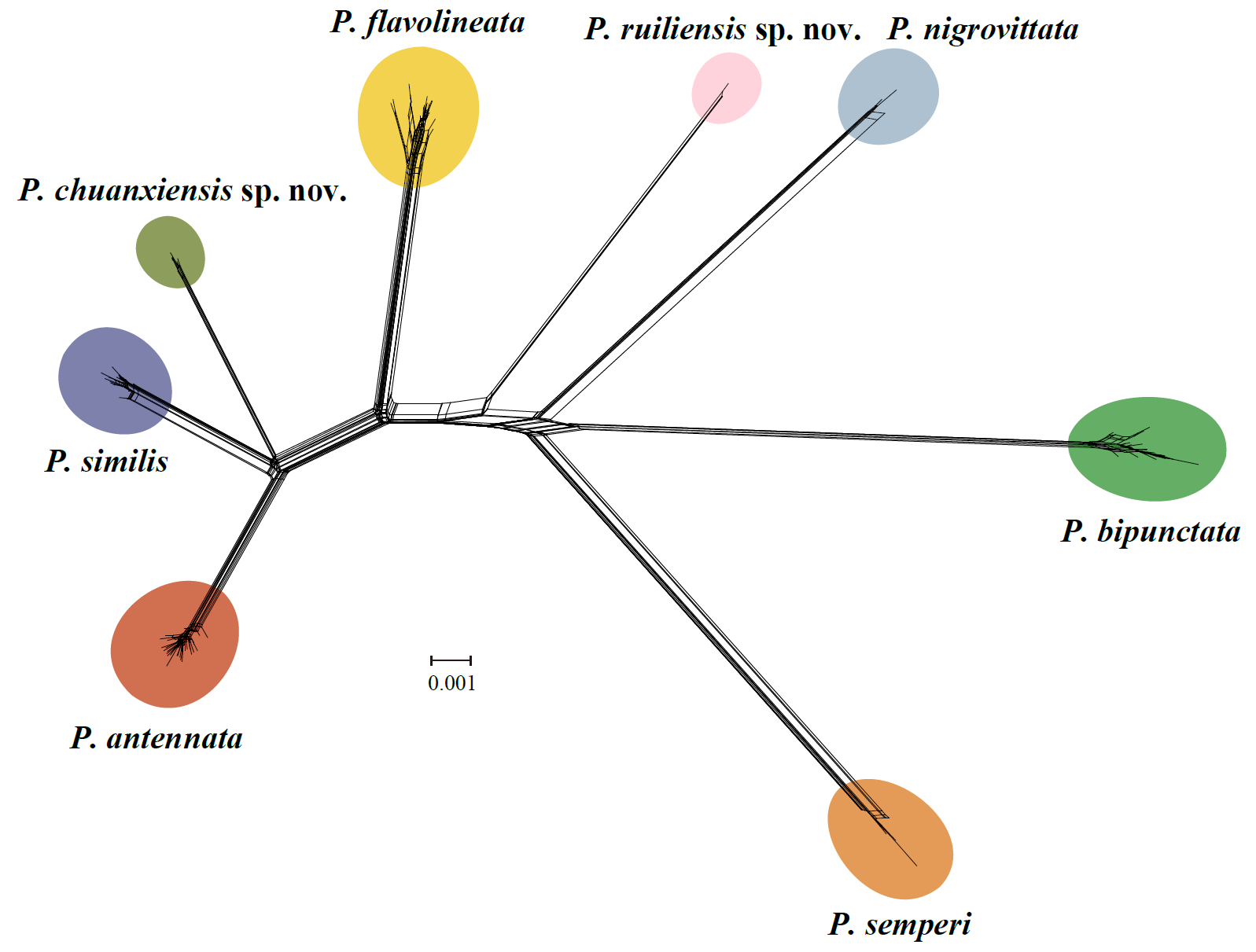


Supplementary Figure 5. Results of structure analysis under K values from 1 to 11. Optimal clustering value for *K* = 3.


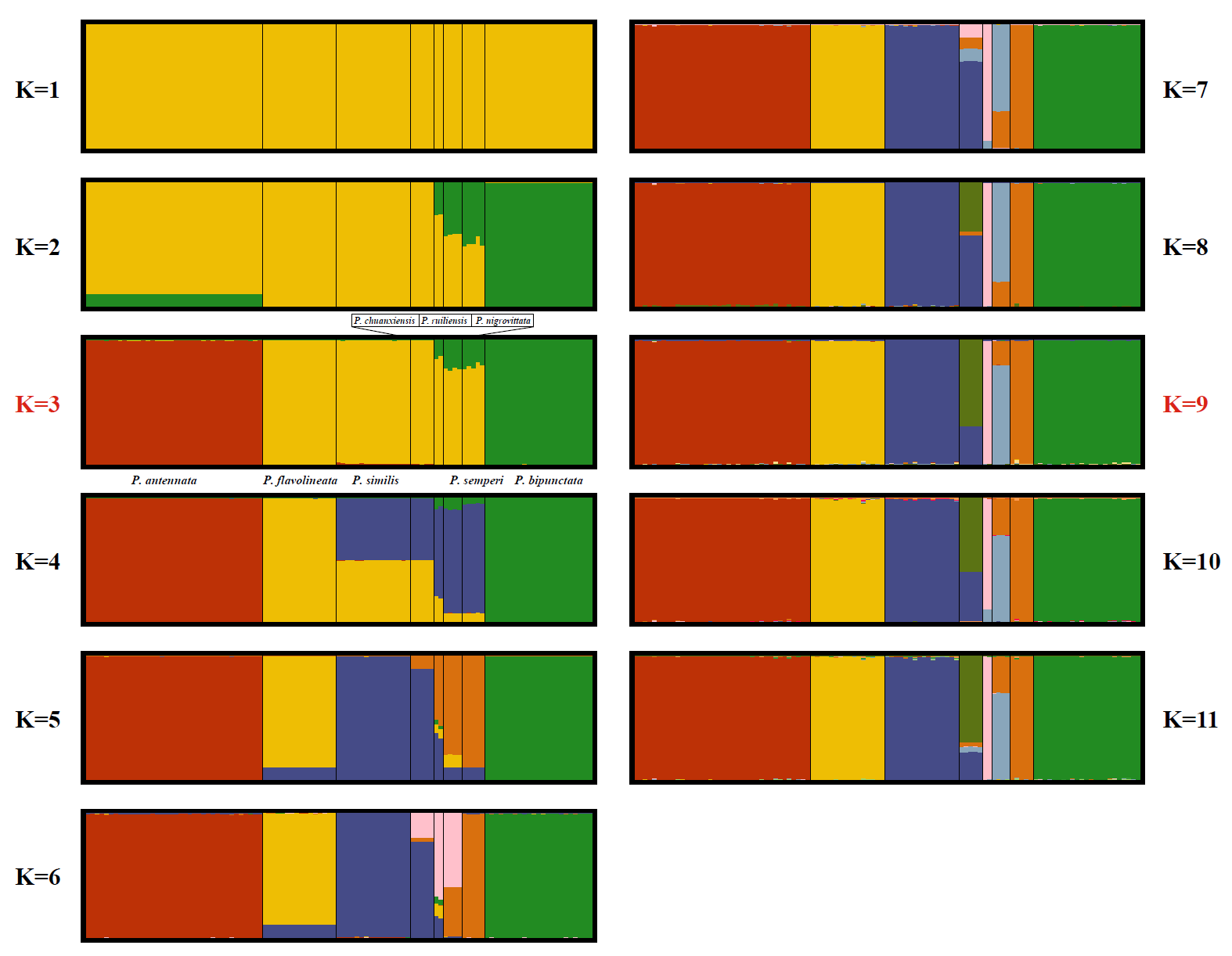


Supplementary Figure 6. Result of Discriminant analysis of principal components (DAPC).


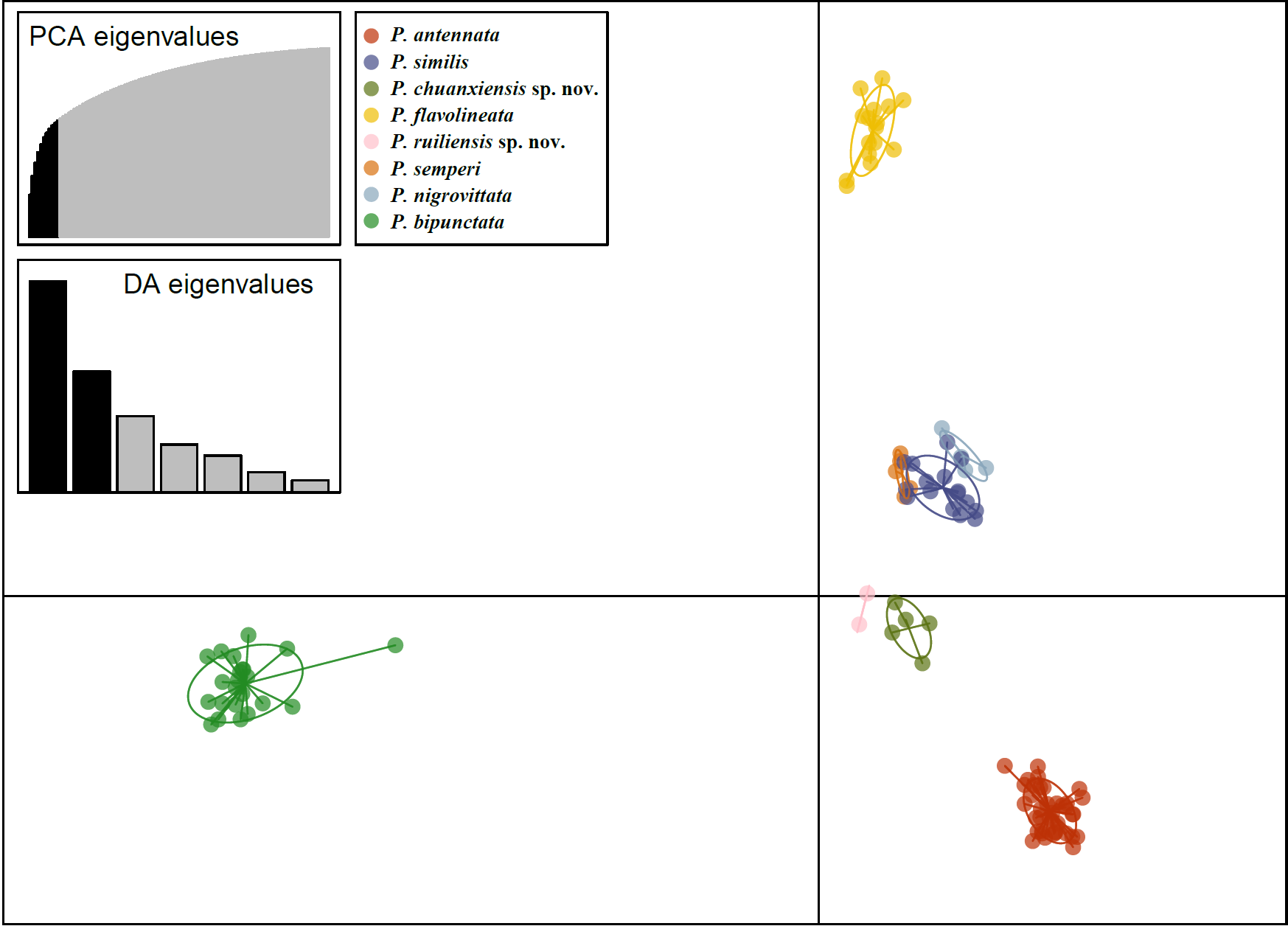


Supplementary Figure 7. Heatmap constructed from estimates of pairwise *Fst* between species.


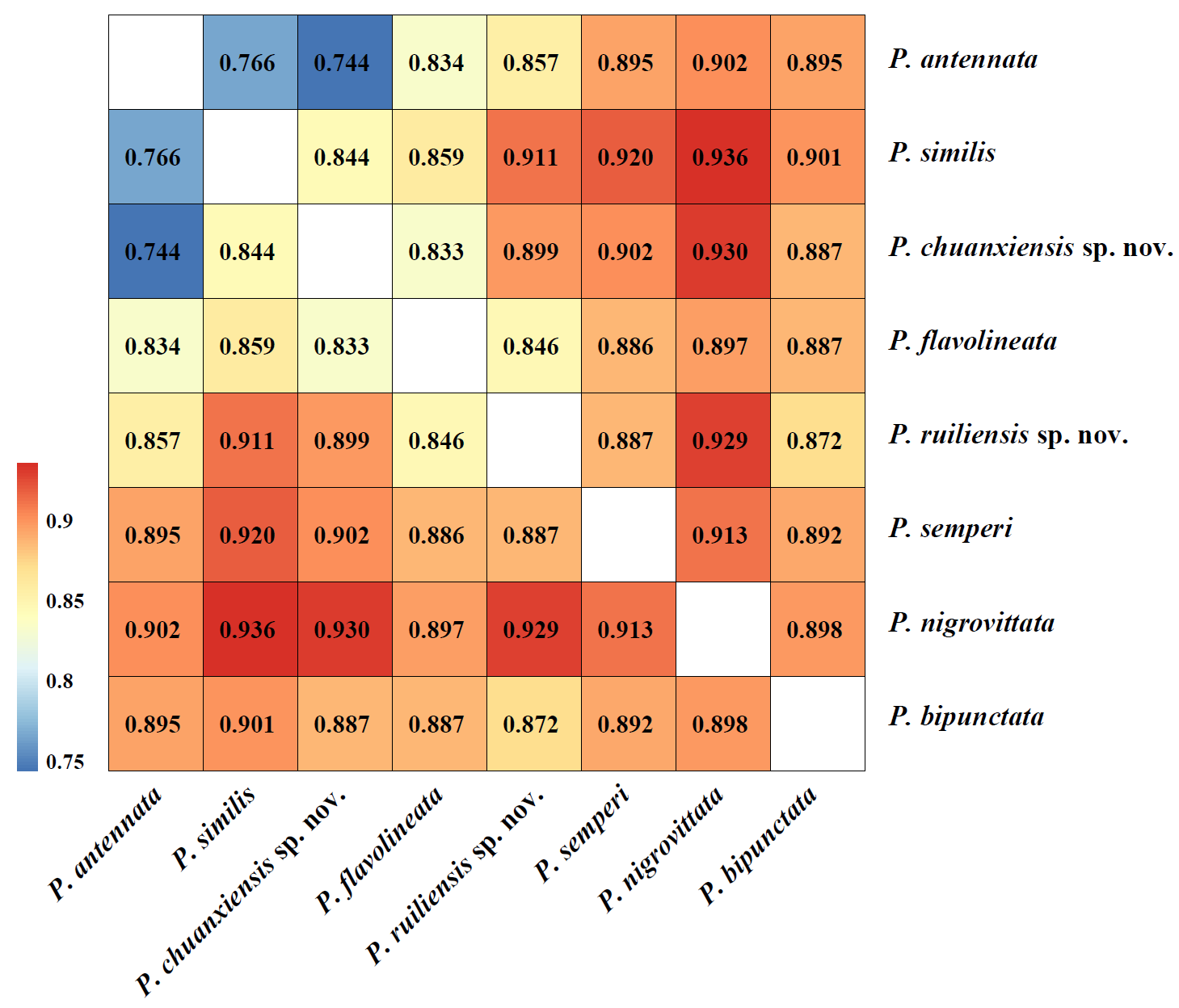


Supplementary Figure 8. Inferred evolutionary relationships from SVDquartets analysis based on SNPs dataset.


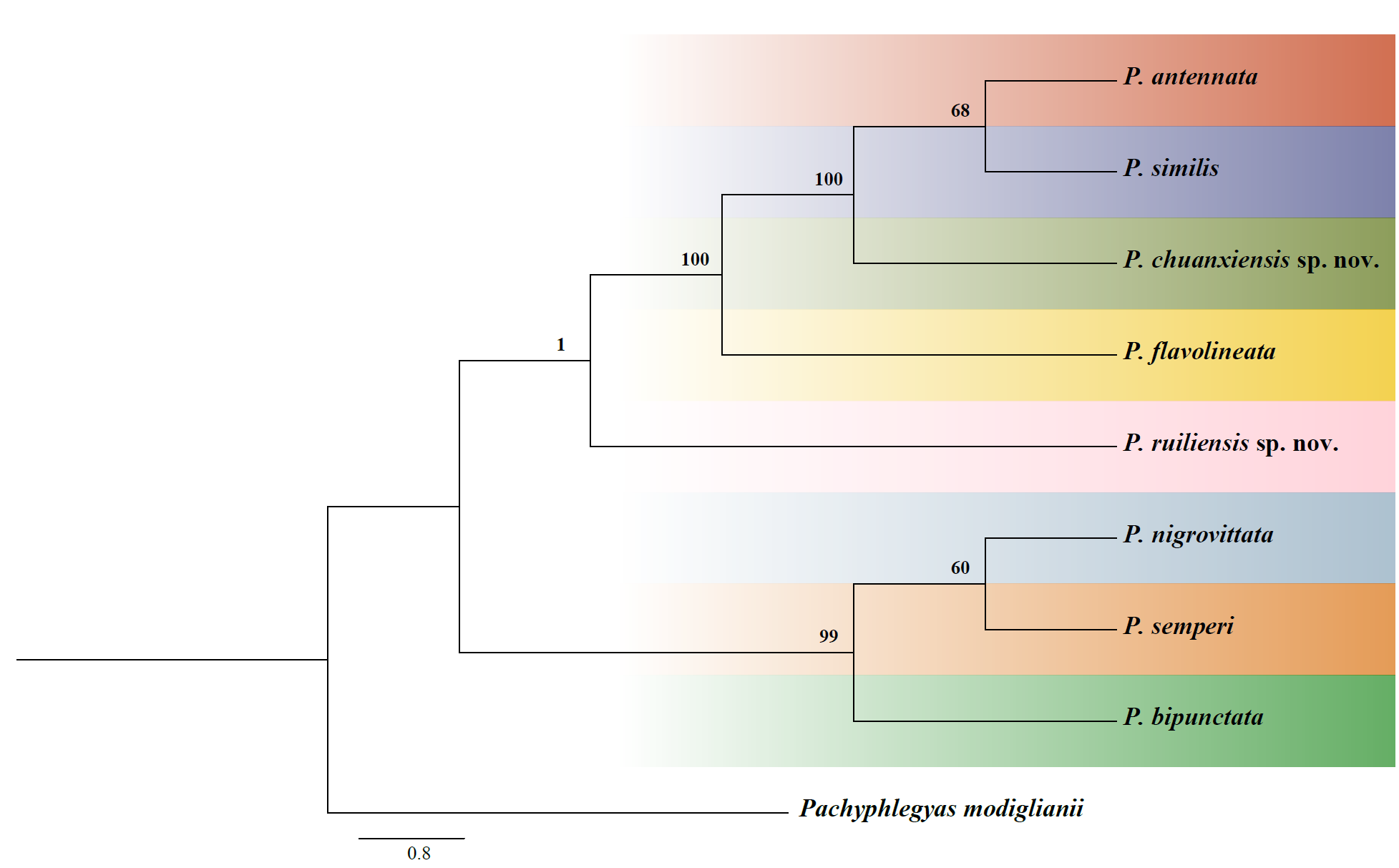


Supplementary Figure 9. Lateral view of head morphology in male *Pachygrontha* species. (**A**) *P. antennata*. (**B**) *P. similis*. (**C**) *P. chuanxiensis* sp. nov. (**D**) *P. flavolineata*. (**E**) *P. ruiliensis* sp. nov. (**F**) *P. nigrovittata*. (**G**) *P. semperi*. (**H**) *P. bipunctata*. The arrows indicate key diagnostic features, including: the depth of the transverse constriction of the pronotum, the morphology of the lateral carina, and the density and distribution of punctation.


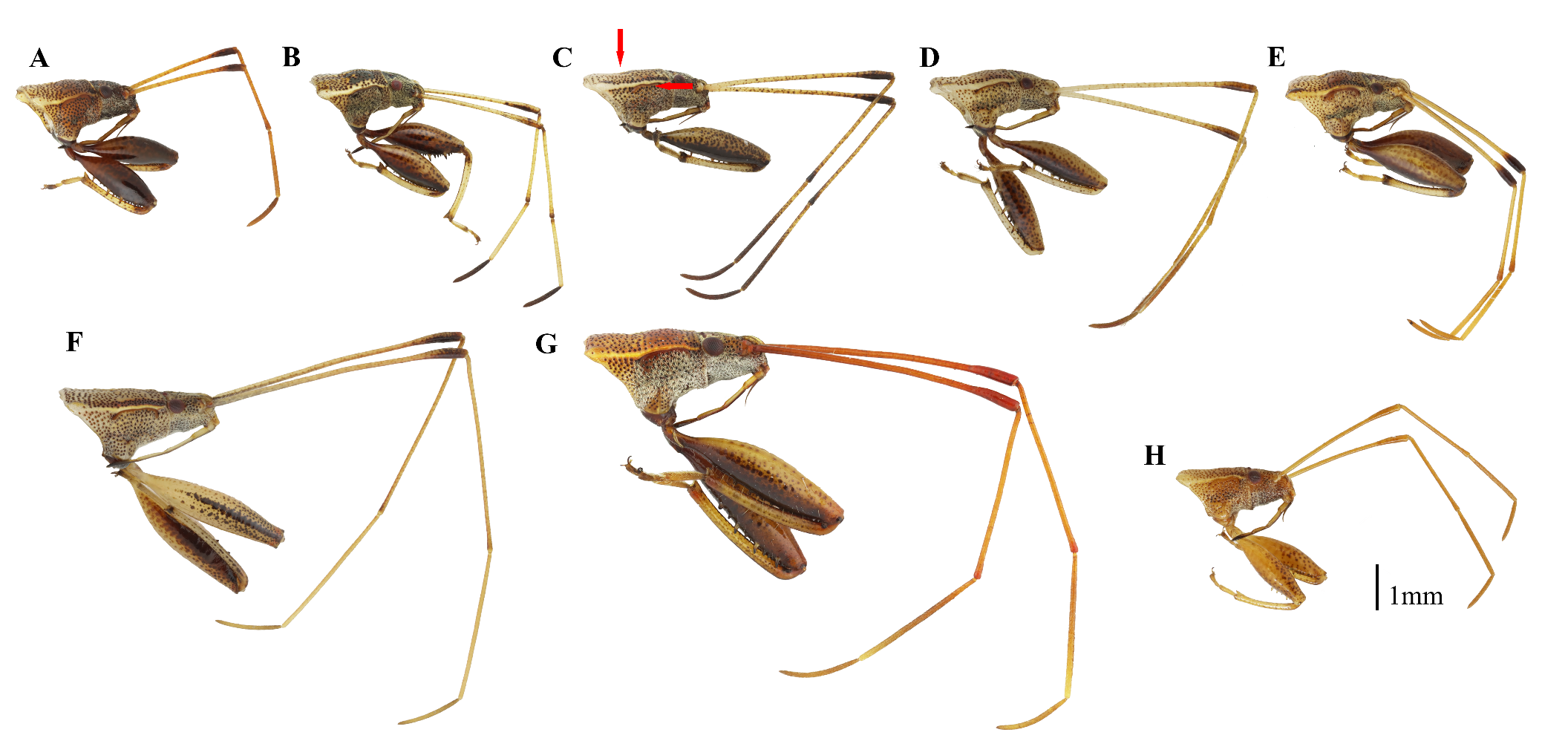


Supplementary Figure 10. Forewing view in male *Pachygrontha* species. (**A**) *P. antennata*. (**B**) *P. similis*. (**C**) *P. chuanxiensis* sp. nov. (**D**) *P. flavolineata*. (**E**) *P. ruiliensis* sp. nov. (**F**) *P. nigrovittata*. (**G**) *P. semperi*. (**H**) *P. bipunctata*. The arrows indicate key diagnostic features, including: the spots at the inner angle, apex, and middle of the apical margin of the corium, as well as the coloration between the veins of the membrane.


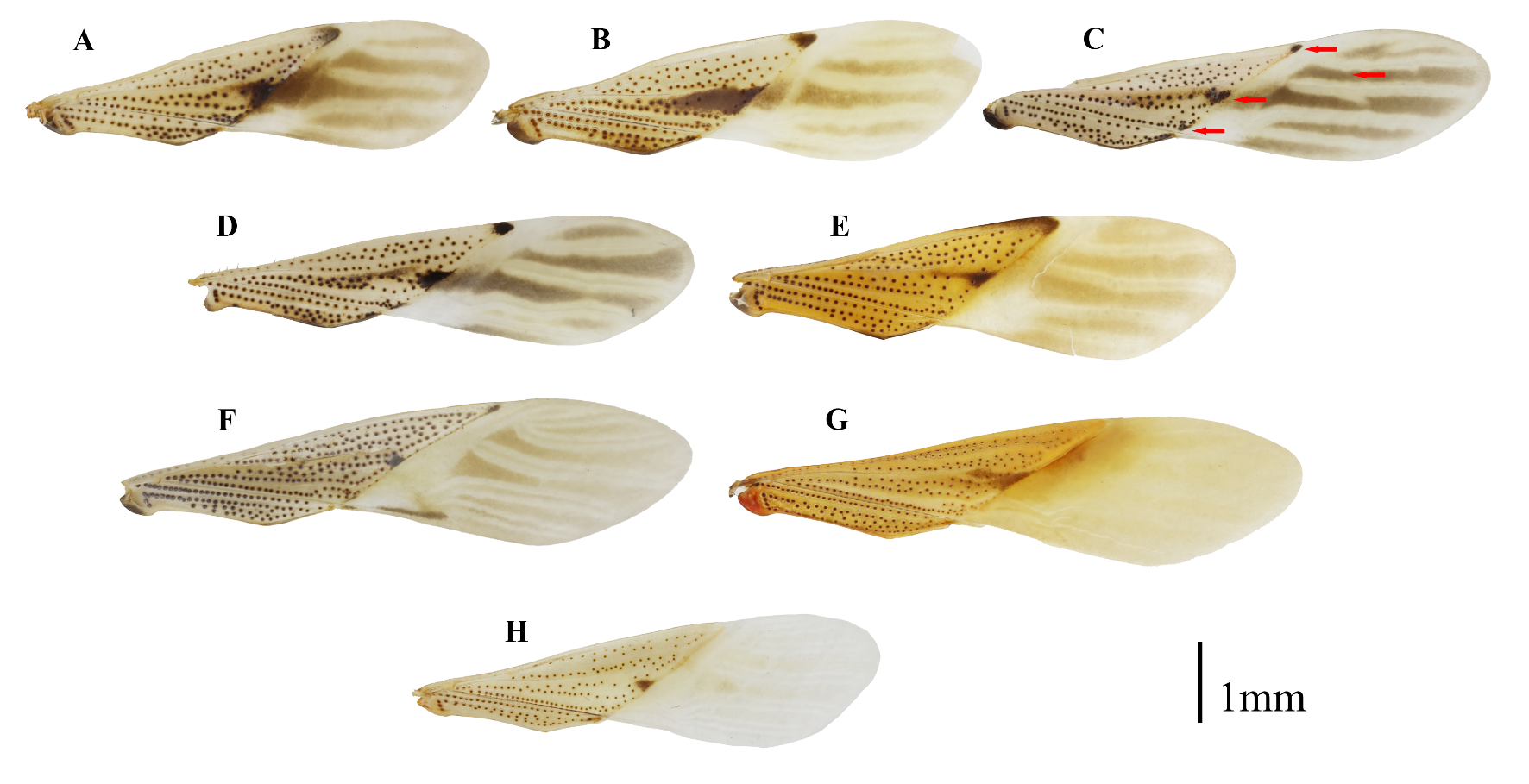


Supplementary Figure 11. Ventral abdominal view of *Pachygrontha* species. (A) *P. antennata*, male. (B) *P. similis*, male. (C) *P. chuanxiensis* sp. nov., male. (D) *P. flavolineata*, male. (E) *P. ruiliensis* sp. nov., male. (F) *P. ruiliensis* sp. nov., female. (G) *P. nigrovittata*, male. (H) *P. semperi*, male. (I) *P. bipunctata*, male. The arrows indicate key diagnostic features, including: black markings on the connexivum adjacent to the apical angle of the corium, coloration of the abdominal venter, and the morphology of the mid-ventral line.


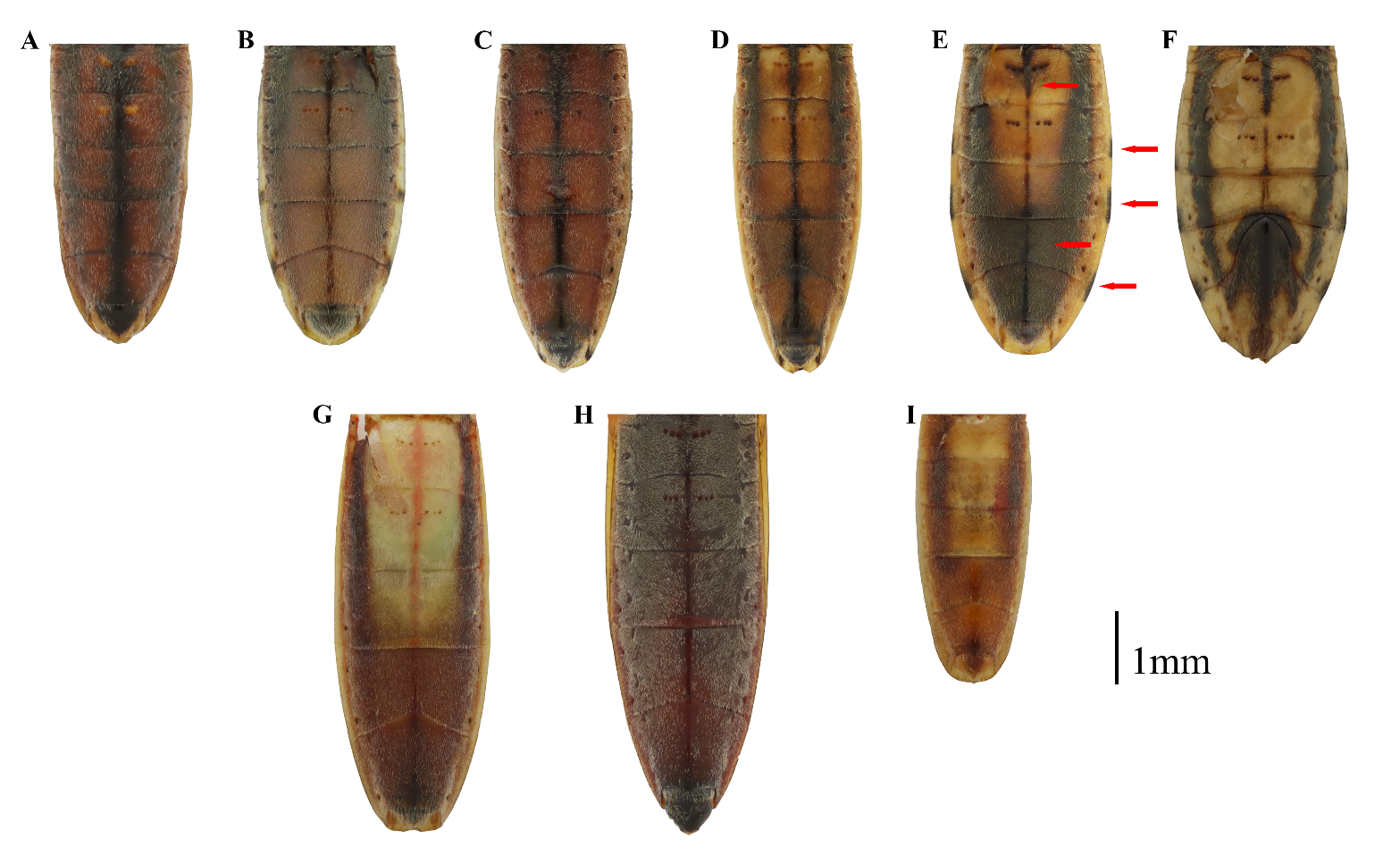

Supplement: Supplementary file 1 — Figure S1: Heatmap constructed from estimates of pairwise mitochondrial genetic distance between species. Figure S2: Results of Principal component analysis (PCA). Figure S3: Phylogenetic tree inferred from the PCG dataset (Left) and PCGR dataset (Right). Values at nodes represent ML bootstrap/BI posterior probability. Figure S4: The phylogenetic networks constructed by the neighbor‐net method based on the SNP dataset. Figure S5: Results of structure analysis under K values from 1 to 11. Optimal clustering value for K = 3. Figure S6: Result of Discriminant analysis of principal components (DAPC). Figure S7: Heatmap constructed from estimates of pairwise F st between species. Figure S8: Inferred evolutionary relationships from SVDquartets analysis based on SNPs dataset. Figure S9: Lateral view of head morphology in male Pachygrontha species. (A) P. antennata . (B) P. similis . (C) P. chuanxiensis sp. nov. (D) P. flavolineata . (E) P. ruiliensis sp. nov. (F) P. nigrovittata . (G) P. semperi . (H) P. bipunctata . The arrows indicate key diagnostic features, including: the depth of the transverse constriction of the pronotum, the morphology of the lateral carina, and the density and distribution of punctation. Figure S10: Forewing view in male Pachygrontha species. (A) P. antennata . (B) P. similis . (C) P. chuanxiensis sp. nov. (D) P. flavolineata . (E) P. ruiliensis sp. nov. (F) P. nigrovittata . (G) P. semperi . (H) P. bipunctata . The arrows indicate key diagnostic features, including: the spots at the inner angle, apex, and middle of the apical margin of the corium, as well as the coloration between the veins of the membrane. Figure S11: Ventral abdominal view of Pachygrontha species. (A) P. antennata , male. (B) P. similis , male. (C) P. chuanxiensis sp. nov., male. (D) P. flavolineata , male. (E) P. ruiliensis sp. nov., male. (F) P. ruiliensis sp. nov., female. (G) P. nigrovittata , male. (H) P. semperi , male. (I) P. bipunctata , male. The arrows indicate key di [file ECE3-16-e73679-s002.docx]
