# Supplementary material for: Integrative Taxonomy of Pachygrontha (Heteroptera: Pachygronthidae) in East and Southeast Asia Reveals New Insights Into Species and Group Delimitation
Source: Ecol Evol. 2026 Jul 1;16(7):e73679. doi: 10.1002/ece3.73679 (PMC13322632; doi:10.1002/ece3.73679)
Supplement: Supplementary file 2 — Table S1: Information on sampling locations for mitochondrial analysis. Table S2: Information on sampling locations for ddRAD analysis. Table S3: Result of STRUCTURE Harvester under K values from 1 to 11. Table S4: Nucleotide polymorphisms in each species based on SNPs dataset. Table S5: Overview of major species groups within the genus Pachygrontha Germar, 1838, showing their distribution ranges and constituent species. Classification follows the system established by Slater (1955). [file ECE3-16-e73679-s001.docx]

**Integrative taxonomy of *Pachygrontha* (Heteroptera: Pachygronthidae) in East and Southeast Asia reveals new insights into species and group delimitation**

Kaibin Wang^1^†, Cuiqing Gao^2^†, Ying Wang^1^, Siying Fu^13^*, Wenjun Bu^1^*

^1^College of Life Sciences, Nankai University, Weijin Road, Nankai, Tianjin 300071, China

^2^Center for Sustainable Forestry in Southern China, College of Forestry and Grassland, Nanjing Forestry University, LongPan Road, Nanjing, Jiangsu 210037, China

^3^School of Synthetic Biology, Research Institute of Applied Biology, College of Life Science, Shanxi University, Taiyuan, Shanxi, 030006 China

†These authors contributed equally to this work.

*Correspondence to be sent to: College of Life Sciences, Nankai University, Tianjin 300071, China; e-mail: [wenjunbu@nankai.edu.cn](mailto:wenjunbu@nankai.edu.cn); [nkufsy@163.com](mailto:nkufsy@163.com)

Supplementary Table 1. Information on sampling locations for mitochondrial analysis.

| Species | Population Codes | Locations | Longitude(E) | Latitude(N) |
| --- | --- | --- | --- | --- |
| *P*. *antennata* | GXGL1 | Guilin, Guangxi, China | 110.473 | 25.874 |
|  | GXGL2 | Guilin, Guangxi, China | 110.473 | 25.874 |
|  | GXGL3 | Guilin, Guangxi, China | 110.473 | 25.874 |
|  | HBXN1 | Xianning, Hubei, China | 114.709 | 29.454 |
|  | HBXN2 | Xianning, Hubei, China | 114.709 | 29.454 |
|  | HBXN3 | Xianning, Hubei, China | 114.709 | 29.454 |
|  | HETS1 | Tangshan, Hebei, China | 118.286 | 40.209 |
|  | HETS2 | Tangshan, Hebei, China | 118.286 | 40.209 |
|  | HETS3 | Tangshan, Hebei, China | 118.286 | 40.209 |
|  | HLSZ1 | Shangzhi, Heilongjiang, China | 127.377 | 45.305 |
|  | HLSZ2 | Shangzhi, Heilongjiang, China | 127.377 | 45.305 |
|  | HLSZ3 | Shangzhi, Heilongjiang, China | 127.377 | 45.305 |
|  | SCYA1 | Yaan, Sichuan, China | 102.749 | 30.728 |
|  | SCYA2 | Yaan, Sichuan, China | 102.749 | 30.728 |
|  | SCYA3 | Yaan, Sichuan, China | 102.749 | 30.728 |
|  | SDYT1 | Yantai, Shandong, China | 121.711 | 37.234 |
|  | SDYT2 | Yantai, Shandong, China | 121.711 | 37.234 |
|  | SNSL1 | Shangluo, Shaanxi, China | 109.866 | 33.007 |
|  | SNSL2 | Shangluo, Shaanxi, China | 109.866 | 33.007 |
|  | SNSL3 | Shangluo, Shaanxi, China | 109.866 | 33.007 |
|  | ZJNB1 | Ningbo, Zhejiang, China | 121.205 | 30.111 |
|  | ZJNB2 | Ningbo, Zhejiang, China | 121.205 | 30.111 |
|  | ZJNB3 | Ningbo, Zhejiang, China | 121.205 | 30.111 |
|  | KR | Daegu, South Korea | 129.111 | 35.889 |
|  | JP* | Mimasaka, Okayama, Japan | 134.249 | 35.092 |
| *P*. *similis* | JXNC | Nanchang, Jiangxi, China | 115.715 | 28.825 |
|  | ZJWZ1 | Wenzhou, Zhejiang, China | 119.937 | 27.351 |
|  | ZJWZ2 | Wenzhou, Zhejiang, China | 119.937 | 27.351 |
|  | CQCK* | Chengkou, Chongqing, China | 108.541 | 31.926 |
| *P*. *chuanxiensis* | SCGZ* | Ganzi, Sichuan, China | 101.862 | 31.150 |
| *P*. *flavolineata* | CQNC1* | Nanchuan, Chongqing, China | 107.210 | 29.121 |
|  | CQNC2* | Nanchuan, Chongqing, China | 107.210 | 29.121 |
|  | HBES* | Enshi, Hubei, China | 108.705 | 30.200 |
|  | YNBS* | Baoshan, Yunnan, China | 98.740 | 24.850 |
|  | YNHH* | Honghe, Yunnan, China | 103.778 | 24.440 |
| *P*. *ruiliensis* | YNHH1* | Honghe, Yunnan, China | 103.482 | 23.256 |
|  | YNHH2* | Honghe, Yunnan, China | 103.482 | 23.256 |
| *P*. *nigrovittata* | GDJM* | Jiangmen, Guangdong, China | 112.928 | 22.235 |
|  | HNSY* | Sanya, Hainan, China | 109.657 | 18.393 |
|  | MAS* | Tapah, Perak, Malaysia | 101.292 | 4.199 |
| *P*. *semperi* | HNSY* | Sanya, Hainan, China | 109.657 | 18.393 |
|  | MAS* | Lenggong, Perak, Malaysia | 100.988 | 5.186 |
| *P*. *bipunctata* | FJNP* | Nanping, Fujian, China | 117.396 | 27.579 |
|  | GDYD* | Yingde, Guangdong, China | 113.134 | 24.485 |
|  | JXJA* | Jian, Jiangxi, China | 115.594 | 26.898 |
|  | MAS* | Sandakan, Sabah, Malaysia | 116.458 | 5.026 |

†The sequenced individuals were derived from previously research (GenBank accession number: PP915831- 915869). *** marked the newly sequenced individuals. Newly sequenced mitogenomes were submitted to GenBank (accession numbers: PX583528 - PX583546).

Supplementary Table 2. Information on sampling locations for ddRAD analysis.

| Species | Population Codes | Locations | Number | Longitude(E) | Latitude(N) |
| --- | --- | --- | --- | --- | --- |
| *P*. *antennata* | HBXN | Xianning, Hubei, China | 10 | 114.709 | 29.454 |
|  | HLSZ | Shangzhi, Heilongjiang, China | 10 | 127.377 | 45.305 |
|  | ZJNB | Ningbo, Zhejiang, China | 10 | 121.205 | 30.111 |
|  | JP | Mimasaka, Okayama, Japan | 8 | 134.249 | 35.092 |
| *P*. *similis* | GZTR | Tongren, Guizhou, China | 3 | 108.18 | 28.66 |
|  | JXGZ | Ganzhou, Jiangxi, China | 3 | 114.46 | 24.54 |
|  | ZJWZ | Wenzhou, Zhejiang, China | 10 | 119.937 | 27.351 |
| *P*. *chuanxiensis* | SCGZ | Ganzi, Sichuan, China | 5 | 101.8624 | 31.1504 |
| *P*. *flavolineata* | CQNC | Nanchuan, Chongqing, China | 1 | 107.2096 | 29.12149 |
|  | HBES | Enshi, Hubei, China | 10 | 108.6936 | 30.1905 |
|  | YNHHA | Honghe, Yunnan, China | 2 | 103.7777 | 24.4396 |
|  | YNHHB* | Honghe, Yunnan, China | 1 | 103.7024 | 24.6999 |
|  | YNBS* | Baoshan, Yunnan, China | 2 | 98.740 | 24.850 |
| *P*. *ruiliensis* | YNHH* | Honghe, Yunnan, China | 2 | 103.482 | 23.256 |
| *P*. *nigrovittata* | GDJM* | Jiangmen, Guangdong, China | 1 | 112.928 | 22.235 |
|  | HNSY* | Sanya, Hainan, China | 2 | 109.657 | 18.393 |
|  | MAS* | Tapah, Perak, Malaysia | 1 | 101.292 | 4.199 |
| *P*. *semperi* | HNSY* | Sanya, Hainan, China | 1 | 109.657 | 18.393 |
|  | MAS* | Lenggong, Perak, Malaysia | 4 | 100.988 | 5.186 |
| *P*. *bipunctata* | FJNPA* | Nanping, Fujian, China | 5 | 117.396 | 27.579 |
|  | FJNPB* | Nanping, Fujian, China | 5 | 117.301 | 27.296 |
|  | GDYD* | Yingde, Guangdong, China | 4 | 113.134 | 24.485 |
|  | JXJA* | Jian, Jiangxi, China | 4 | 115.594 | 26.898 |
|  | MAS* | Sandakan, Sabah, Malaysia | 5 | 116.458 | 5.026 |
| *Pachyphlegyas modiglianii* | YNDH* | Dehong, Yunnan, China | 2 | 97.8306 | 24.0663 |
|  | HNBS* | Baisha, Hainan, China | 2 | 109.418 | 19.077 |

†The sequenced individuals were derived from previously research (GenBank accession number: PRJNA1274209, PRJNA1122967). *** marked the newly sequenced individuals. Newly sequenced ddRAD data are available in the NCBI SRA (**PRJNA1359458**) and identified by their unique population codes.

Supplementary Table 3. Result of STRUCTURE Harvester under K values from 1 to 11.

| K | Reps | Mean LnP(K) | Stdev LnP(K) | Ln'(K) | \|Ln''(K)\| | Delta K |
| --- | --- | --- | --- | --- | --- | --- |
| 1 | 10 | -11459.3 | 0.1075 | NA | NA | NA |
| 2 | 10 | -7371.06 | 418.8402 | 4088.28 | 1641.25 | 3.918559 |
| 3 | 10 | -4924.03 | 0.1889 | 2447.03 | 1400.38 | 7415.059 |
| 4 | 10 | -3877.38 | 129.7552 | 1046.65 | 194.67 | 1.500286 |
| 5 | 10 | -3025.4 | 237.5718 | 851.98 | 450.3 | 1.895427 |
| 6 | 10 | -2623.72 | 268.3693 | 401.68 | 142.49 | 0.530947 |
| 7 | 10 | -2364.53 | 182.3019 | 259.19 | 229.49 | 1.258846 |
| 8 | 10 | -2334.83 | 297.6998 | 29.7 | 61.27 | 0.205811 |
| 9 | 10 | -2243.86 | 194.1135 | 90.97 | 204.72 | 1.054641 |
| 10 | 10 | -2357.61 | 235.1902 | -113.75 | 109.72 | 0.466516 |
| 11 | 10 | -2361.6400 | 182.0882 | -4.030000 | NA | NA |

Supplementary Table 4. Nucleotide polymorphisms in each species based on SNPs dataset.

| Species | Obs. Het-mean | Obs. Het-s.d. | Exp. Het-mean | Exp. Het-s.d. | Theta (*π_S_*) | *F* |
| --- | --- | --- | --- | --- | --- | --- |
| *P*. *antennata* | 0.08400 | 0.10943 | 0.10571 | 0.14362 | 0.017164 | 0.880455 |
| *P*. *similis* | 0.09973 | 0.09670 | 0.14791 | 0.16767 | 0.016280 | 0.897225 |
| *P*. *flavolineata* | 0.09637 | 0.09262 | 0.16368 | 0.15088 | 0.026240 | 0.8923125 |
| *P*. *chuanxiensis* | 0.32844 | 0.19153 | 0.36555 | 0.14608 | 0.006660 | 0.9494 |
| *P*. *ruiliensis* | 0.48206 | 0.24132 | 0.54260 | 0.07785 | 0.022345 | 0.8622 |
| *P*. *nigrovittata* | 0.27647 | 0.15634 | 0.32647 | 0.11799 | 0.015382 | 0.90335 |
| *P*. *semperi* | 0.22696 | 0.16231 | 0.30676 | 0.12648 | 0.019637 | 0.88794 |
| *P*. *bipunctata* | 0.09069 | 0.10003 | 0.11362 | 0.12313 | 0.021458 | 0.865813 |

†Obs. Het, observed heterozygosity; Exp. Het, expected heterozygosity; Theta (*π_S_*), nucleotide diversity; F, inbreeding coefficient.

Supplementary Table 5. Overview of major species groups within the genus ***Pachygrontha* Germar, 1838**, showing their distribution ranges and constituent species. Classification follows the system established by Slater (1955).

| Species group | Distribution ranges | Constituent species |
| --- | --- | --- |
| *the bakeri group** | Large islands surrounding the continental Asia, including the Philippines, extending to New Guinea, Borneo, and the Eastern Indian Islands and Malaya. | *P*. *miriformis*, *P*. *carinata*, *P*. *bakeri bakeri*, *P*. *bakeri gracilis*,  *P*. *harrisi*, *P*. *semperi**, *P*. *longicornis*, *P*. *vidua* |
| *the bipunctata group** | Tropical regions of the Old World, encompassing the Oriental and Afrotropical regions, including Madagascar. | *P*. *bipunctata bipunctata**, *P*. *bipunctata incipiens*, *P*. *congoensis*,  *P*. *quadripunctata* |
| *the nigrovittata group** | Oriental and Afrotropical regions. | *P*. *lestoni*, *P*. *nigrovittata** |
| *the antennata group** | The easternmost part of the Palearctic region. | *P*. *antennata antennata**, *P*. *antennata nigriventris**, *P*. *similis** |
| *the lurida group* | The Philippines and Yakushima Island, Japan. | *P*. *lurida lurida*, *P*. *lurida yakuensis*, *P*. *angusta* |
| *the lineata group* | Southern Africa and Mauritius. | *P*. *lineata*, *P*. *paralineata*, *P*. *pseudolineata* |
| *the longiceps group* | Tropical South America, Central America, and parts of the West Indies. | *P*. *barberi*, *P*. *grossa*, *P*. *longiceps*, *P*. *minarum minarum*,  *P*. *minarum saileri* |
| *the oedancalodes group* | Tropical South America, Central America, and parts of the West Indies. | *P*. *oedancalodes oedancalodes*, *P*. *oedancalodes carvalhoi*,  *P*. *compacta* |
| No species group |  | *P*. *robusta*, *P*. *walkeri*, *P*. *africana*, *P*. *lewisi*, *P*. *austrina*,  *P*. *flavolineata**, ***P***. ***ruiliensis* sp. nov.** *, ***P***. ***chuanxiensis* sp. nov.** * |

†Species groups and species marked with * are included in this study.
